# Functional Centromeres Determine the Activation Time of Pericentric Origins of DNA Replication in *Saccharomyces cerevisiae*

**DOI:** 10.1371/journal.pgen.1002677

**Published:** 2012-05-10

**Authors:** Thomas J. Pohl, Bonita J. Brewer, M. K. Raghuraman

**Affiliations:** 1 Molecular and Cellular Biology Program, University of Washington, Seattle, Washington, United States of America; 2 Department of Genome Sciences, University of Washington, Seattle, Washington, United States of America; University of Medicine and Dentistry of New Jersey–New Jersey Medical School, United States of America

## Abstract

The centromeric regions of all *Saccharomyces cerevisiae* chromosomes are found in early replicating domains, a property conserved among centromeres in fungi and some higher eukaryotes. Surprisingly, little is known about the biological significance or the mechanism of early centromere replication; however, the extensive conservation suggests that it is important for chromosome maintenance. Do centromeres ensure their early replication by promoting early activation of nearby origins, or have they migrated over evolutionary time to reside in early replicating regions? In *Candida albicans*, a neocentromere contains an early firing origin, supporting the first hypothesis but not addressing whether the new origin is intrinsically early firing or whether the centromere influences replication time. Because the activation time of individual origins is not an intrinsic property of *S. cerevisiae* origins, but is influenced by surrounding sequences, we sought to test the hypothesis that centromeres influence replication time by moving a centromere to a late replication domain. We used a modified Meselson-Stahl density transfer assay to measure the kinetics of replication for regions of chromosome XIV in which either the functional centromere or a point-mutated version had been moved near origins that reside in a late replication region. We show that a functional centromere acts in *cis* over a distance as great as 19 kb to advance the initiation time of origins. Our results constitute a direct link between establishment of the kinetochore and the replication initiation machinery, and suggest that the proposed higher-order structure of the pericentric chromatin influences replication initiation.

## Author Summary

Genome duplication requires the orderly initiation of DNA synthesis at sites called origins of replication. It has long been known that different origins become active at different times in S-phase (the period during which cells duplicate their chromosomes). Although such temporal regulation of replication is broadly conserved among eukaryotes, how this regional control of replication time occurs largely remains a mystery. The early replication of baker's yeast centromeres (genetic elements essential for proper segregation of chromosomes during cell division) is one frequently cited example of temporal regulation, yet the biological significance of early centromere replication also remains speculative. Increasing evidence suggests that early centromere replication is a conserved feature of the DNA replication program across many species. Here, we show that centromeres in this yeast can advance the time at which origins in their genomic neighborhood initiate DNA replication. The distance over which centromeres can influence origin activation time extends up to 19 kilobases. We further show that centromere-mediated early origin activation depends on the centromere's ability to recruit at least a subset of the proteins needed for chromosome segregation. This study thus provides the first direct functional link between kinetochore establishment and the mechanisms of DNA replication initiation.

## Introduction

Centromere function, the ability to assemble a kinetochore and mediate chromosome segregation during meiosis and mitosis, is critical for the survival and propagation of eukaryotic organisms. Defects in centromere/kinetochore complexes lead to genome instability and susceptibility to cancer and cell death [1]–[3]. Therefore, it is important that properly functioning centromeres be established on both sister chromatids following replication of centromeric DNA and prior to the initiation of chromosome segregation. Centromeres in the budding yeast *Saccharomyces cerevisiae* replicate early in S-phase [4]–[8] and increasing evidence suggests that early centromere replication is conserved among fungi and is prevalent for at least a subset of centromeres in higher eukaryotes [9]–[13]. Yet surprisingly little is known about the mechanism that accomplishes this early replication. It has been hypothesized that early replication of centromere DNA provides sufficient time for the centromere-specific histone, CenH3, to be incorporated on both sister chromatids and to ensure subsequent microtubule attachment [14]–[16]. Consistent with this idea, Feng et al. showed that a delay in centromere DNA replication in the absence of the replication checkpoint leads to increased aneuploidy [16]. It is thought that this observed increase in aneuploidy is due to the lack of properly bi-oriented sister chromatids.

Currently, it is unclear if centromeres play an active role in their own early replication by influencing activation of nearby origins of replication (origins). An alternative possibility is that centromeres have migrated over evolutionary time to reside in early replicating regions of the genome.

Indirect evidence supporting the idea that centromeres play an active role in their own early replication comes from a study examining epigenetic inheritance of centromeres in the pathogenic yeast *Candida albicans* [11]. The centromeres of *C. albicans* are considered regional centromeres akin to those of higher eukaryotes because they are not defined by any distinct DNA sequence. Instead, regional centromeres are defined by broad stretches of repetitive DNA sequences ranging from about 3 kilobases (kb) in *C. albicans* to megabases in humans. Regional centromeres that have been analyzed for replication time have been shown to contain origins within them [9], [10], [17]. Koren et al. showed that the *de novo* formation of an early activated origin within the neocentromeric region is responsible for the early replication of spontaneously formed neocentromeres in *C. albicans* [11]. The authors further showed that the origin recognition complex (ORC), which is essential for origin activation, is recruited to the neocentromere. Thus, centromeres of *C. albicans* appear to recruit at least a subset of the required replication initiation machinery. However, it is unclear from these studies whether centromere function directly influences origin activation time or if these regions merely provide a favorable environment for ORC recruitment, with early origin activation time being determined independently of centromere function.

Distinguishing between these possibilities is difficult because no distinct sequences for either centromeres or origins have been identified in *C. albicans*. Therefore, the function of the centromere responsible for replication initiation cannot effectively be separated from the kinetochore-binding portion of the centromere. For example, removal of centromere DNA in *C. albicans* results in removal of the origins contained within that sequence. Therefore, determining if centromeres regulate origin activation time requires a situation in which the activity of the origin responsible for centromere replication can be separated from the centromere. The small sizes and well-defined sequences of centromeres and origins in *S. cerevisiae* provide us the opportunity to answer this question.

The origins of *S. cerevisiae* have been extensively studied and are defined by an 11 base pair (bp) consensus sequence that is necessary but not sufficient to initiate DNA synthesis [18]. Unlike *C. albicans*, the centromeres in *S. cerevisiae* are small, spanning only 125 bps and do not, with one possible exception (*CEN3*), contain potential origins [6], [19]–[21]. However, all of the centromeres of *S. cerevisiae* reside in early replicating portions of the genome, suggesting that centromeres play a role in regulating origin activation time.

Early replication is not restricted to portions of the genome immediately flanking centromeres. In fact, early and late replicating blocks of DNA are interspersed throughout the genome [7], [8]. The patterns in which temporal blocks are arranged indicate that origins can be crudely grouped into four classes with respect to their replication time and chromosomal position: centromere-proximal early, noncentromere-proximal early, telomere-proximal late, and nontelomere-proximal late [6]–[8]. Telomeres delay the activation times of nearby origins while late activation of non-telomeric origins is determined by an unknown mechanism involving unspecified DNA sequences located up to 14 kb away from the origin [22], [23]. Plasmids containing the minimum required sequence for origin activation tend to replicate early, suggesting that early activation is the default state for origins [24]. However, in a plasmid construct, a DNA element immediately downstream of the *URA3* gene can advance origin activation time of an adjacent copy of *ARS1* through unknown means [25], [26]. In light of these data, we hypothesize that there is no common “default” activation time and that centromeres act as one of the determinants for early origin activation.

By measuring replication time locally as well as genome-wide in a strain with an ectopic centromere, we show that centromeres in *S. cerevisiae* act in *cis* to promote early activation on origins positioned as far as 19 kb away. Our results suggest that centromere-dependent early origin activation has a gradient effect such that the closer the origin is to the centromere the more profound is the timing effect on that origin. In addition, we show for the first time that early activation of centromere proximal origins is dependent on centromere function, suggesting that the ability of centromeres to establish proper kinetochore-to-microtubule attachments is important for regulating origin initiation.

## Results

### Proximity to centromeres influences time of origin activation

**Figure 1:**
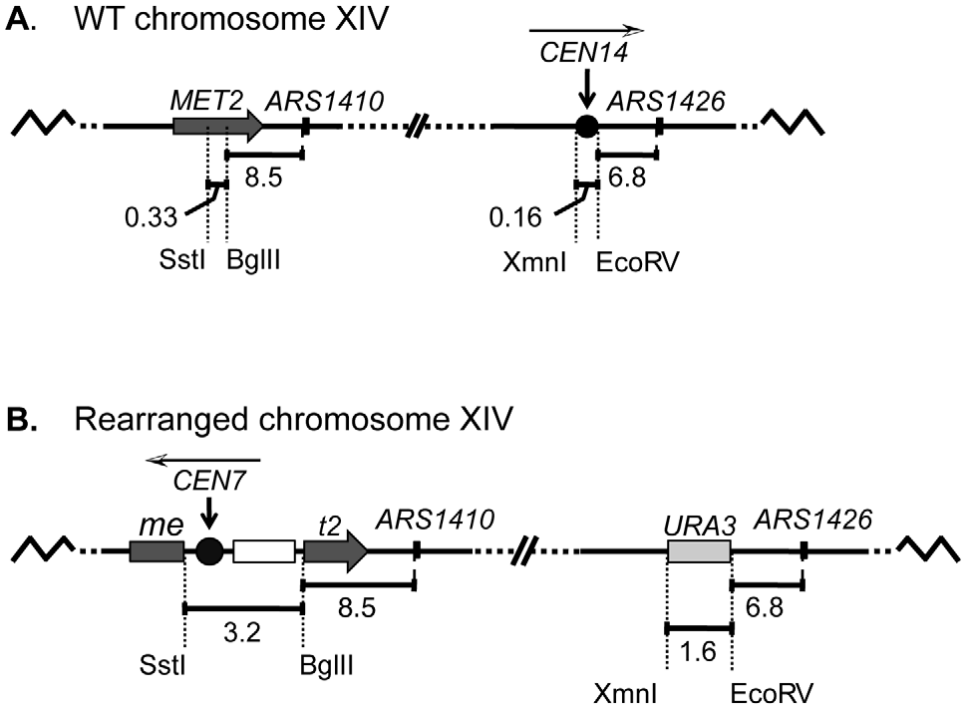
A schematic diagram of chromosome XIV in wild-type (WT) and rearranged strains. (A) In the WT strain, the BglII restriction site in MET2 is located 8.5 kb to the left of ARS1410. Centromere XIV resides in its endogenous position located 6.8 kb to the left of ARS1426. (B) In the rearranged strain the endogenous centromere was replaced with a URA3 selectable marker while a functional centromere was integrated along with LEU2 (open box) into the MET2 locus such that the centromere was positioned ∼11.5 kb from ARS1410. The white and black arrowhead above each centromere indicates the direction of the centromere DNA elements CDEI, CDEII, CDEIII.

*S. cerevisiae* centromeres lie close to origins that undergo replication in early S-phase. To test the hypothesis that centromeres contribute to the early activation of these adjacent origins, we obtained a strain in which the centromere on chromosome XIV (Figure 1A) had been relocated to a distal location on the left chromosomal arm [27] (Figure 1B). At this new location the centromere is positioned in a late replicating region near the potential origin *ARS1410*. We reasoned that if centromeres can influence origin activation time, then the origins that are closest to the moved centromeres would have the greatest chance of being affected.

**Figure 2:**
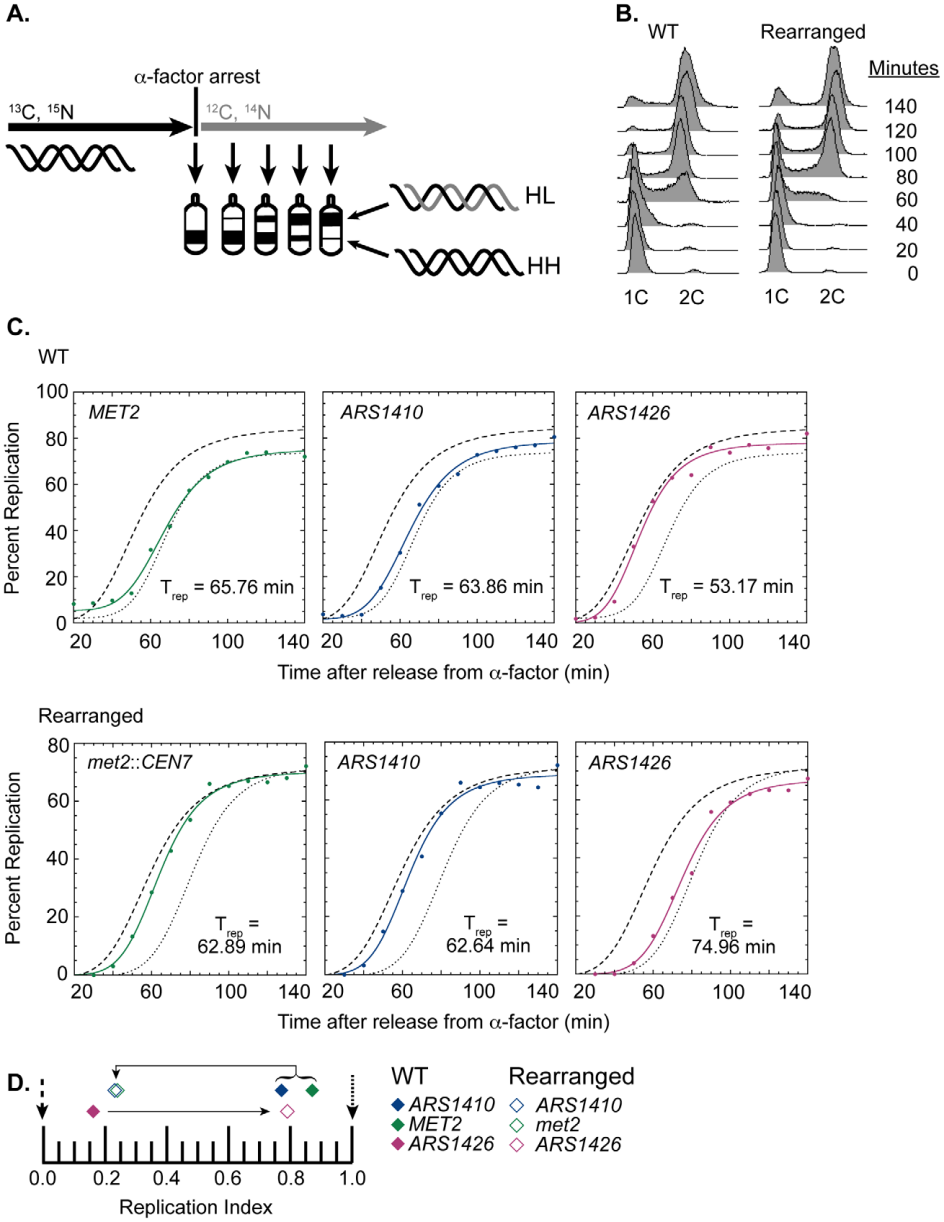
Replication time of native and relocated centromeres on chromosome XIV. (A) Cartoon depiction of experimental setup. Cells were grown in medium containing heavy carbon (^13^C) and nitrogen (^15^N) isotopes. Upon genome saturation with the heavy isotopes, cells were arrested by the addition of alpha factor and released synchronously in medium containing light carbon (^12^C) and nitrogen (^14^N) isotopes. The cells were then collected over the next 140 minutes and their DNA was extracted, digested with EcoRI, and separated via ultra centrifugation in cesium chloride gradients such that unreplicated DNA resides lower in the gradient than newly replicated DNA. DNA samples were then collected and analyzed through drip fractionation. (B) S phase progression of WT (left) and rearranged (right) cells as measured by flow cytometry. Cells from both strains entered S-phase by 40 minutes and achieved 2C DNA content by 140 minutes as indicated by the peak shift from 1C to 2C DNA content. (C) Replication kinetic curves for met2 or MET2, ARS1410, and ARS1426 in WT (top panel) and rearranged (bottom panel) cells. The kinetic curves for ARS306 and R11 are shown as dashed and dotted lines, respectively. T_rep_ is the time of half-maximal replication for each locus (see Materials and Methods). (D) Replication indices for met2 or MET2 (green), ARS1410 (blue), and ARS1426 (magenta) in WT (solid diamonds) and rearranged (empty diamonds) strains. ARS306 (black dashed arrow) and R11 (black dotted arrow) were used as early and late timing standards, respectively. In the WT strain, MET2, ARS1410, and ARS1426 had replication indices of 0.87, 0.77, and 0.16 respectively. In the rearranged strain, met2, ARS1410, and ARS1426 had replication indices of 0.24, 0.23, and 0.79, respectively. Direction of the black arrows indicates the direction of the shift in replication index for each locus between WT and rearranged strains.

The replication kinetics of three loci on chromosome XIV (*met2* or *MET2*, *ARS1410*, and *ARS1426*) in the rearranged and wild type (WT) strains were examined using a modified version of the Meselson-Stahl density transfer experiment [4]. Haploid cells grown in the presence of dense ^13^C and ^15^N isotopes were arrested prior to S-phase. The cells were synchronously released into medium containing isotopically light carbon and nitrogen, and samples were collected at various times during the ensuing S-phase. Newly synthesized DNA was composed of light isotopes resulting in replicated DNA being hybrid or heavy-light (HL) in density whereas unreplicated DNA remained heavy-heavy (HH) in density. DNA was extracted from each cell sample, digested with restriction enzyme EcoRI, and subjected to ultracentrifugation in cesium chloride gradients. The gradients were then drip fractionated, and the kinetics with which the EcoRI restriction fragments containing each of the three loci of interest shifted from heavy to hybrid density were compared via slot blot analysis (Figure 2A; also see Materials and Methods).

WT cells entered S-phase by 40 minutes after release from alpha factor arrest, and most of the cells reached 2C DNA content between 120 and 140 minutes (Figure 2B). The percent replication of *MET2*, *ARS1410*, and *ARS1426* genomic fragments was calculated for each sample and plotted with respect to time (Figure 2C). The time of replication (T_rep_) for each locus was calculated as the time it reached half maximal replication (see Materials and Methods). *ARS306*, one of the earliest known origins, and R11, a late replicating fragment on chromosome V, were used as timing standards for comparison. To facilitate comparison between cultures, these T_rep_ values were converted to replication indices [22] by assigning *ARS306* a replication index (RI) of 0 and R11 an RI of 1.0. Most other genomic loci have RIs between 0 and 1.0. The T_rep_ values for *MET2*, *ARS1410*, and *ARS1426* were then converted to RIs corresponding to the fraction of the *ARS306*-R11 interval elapsed when the T_rep_ for each locus was obtained. As previously observed, *MET2* replicated late in the WT strain (RI = 0.87), as did its nearest ARS, *ARS1410* (RI = 0.77), while *ARS1426* replicated early (RI = 0.16) (Figure 2C and 2D).

Similar to WT cells, the cells with the relocated centromere entered S-phase by 40 minutes following release from alpha factor arrest and reached 2C DNA content between 120 and 140 minutes (Figure 2B). In contrast to the WT strain, both *met2* and *ARS1410* replicated early with respective RIs of 0.24 and 0.23 (Figure 2C and 2D). Consistent with *ARS1410* being the origin from which *met2* replicates, *ARS1410* maintained a slight timing advantage over *met2*. Meanwhile, *ARS1426* became later replicating (RI = 0.79) in the absence of its nearby centromere (Figure 2C and 2D). Similar results were obtained using an independent segregant (see Figure S1).

**Figure 3:**
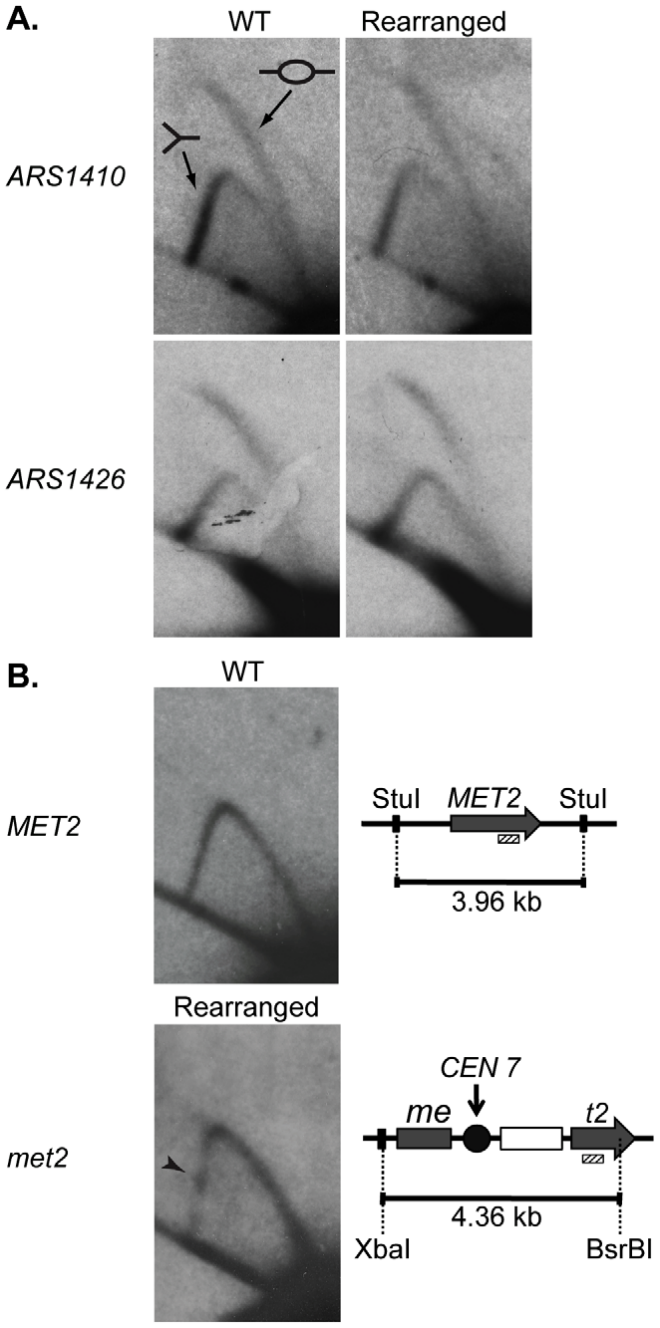
2D gel analysis of ARS1410, ARS1426, and MET2 and met2. DNA fragments containing a functional origin are detected as a bubble arc (depicted by the bubble fragment) while fragments that are passively replicated are detected as a Y-arc (depicted as a Y shaped fragment). (A) ARS1410 and ARS1426 are functional origins in the WT and rearranged strains. (B) 2D gel analysis of the MET2 or met2 locus in the WT and the rearranged strains. WT DNA was digested with the restriction enzyme StuI giving a fragment of 3.96 kb centered on MET2 (grey arrow). Rearranged DNA was double digested with XbaI and BsrBI resulting in a 4.36 kb fragment harboring most of met2, the integrated centromere (black circle), and the LEU2 marker (white rectangle). The absence of a bubble arc when probed for the 3′ end of MET2 and met2 (hashed rectangle) indicates that an origin is not present on either DNA fragment. The centromere in the rearranged construct was detected as a pause site (black arrowhead) visualized as a dot of relatively increased intensity on the descending Y-arc.

There are three explanations for the change in replication time of the *met2* locus: (1) the centromere advances the time of activation of *ARS1410*; (2) the centromere increases the efficiency (percentage of cells in which an origin is active) of *ARS1410*; and/or (3) insertion of the centromere created a new origin at the site. To distinguish among these possibilities we examined the replication of *ARS1410*, *ARS1426*, and *met2* or *MET2* by two-dimensional (2D) gel electrophoresis [28]. The presence of bubble-arcs indicated that *ARS1410* is indeed a functional origin in both the WT and rearranged strains (Figure 3A). Highly efficient origins display a more intense bubble-arc signal relative to the Y-arc [29]. Based on the similarity of bubble- to Y-arc ratios we conclude that the centromere has no obvious effect on the efficiency of *ARS1410* (Figure 3A). A similar result was obtained for *ARS1426* (Figure 3A) supporting the idea that the observed timing changes are not due to changes in efficiency of existing origins. No bubble-arc was observed for the *MET2* locus in the WT strain or the *met2* locus in the rearranged strain (Figure 3B), indicating that an origin was not created by insertion of the centromere into the *MET2* locus. Together these data suggest that the presence of a nearby centromere or some feature of its pericentric DNA induces early activation of origins.

### Centromere function is required for early activation of nearby origins

DNA sequences that determine origin activation time have remained largely elusive. However, previous work has shown that sequences flanking a subset of origins on chromosome XIV delay the activation of those origins [22]. These sequences, coined “delay elements”, can reside up to 14 kb from their affected origins. Therefore, it is possible that by integrating the centromere into the *MET2* locus in the rearranged strain, a delay element responsible for making *ARS1410* late activating was disrupted or pushed out of its effective range, thereby causing the origin to fire early. Although this scenario would explain the change in the replication times of *met2* and *ARS1410*, the observation that *ARS1426* became later replicating when the centromere was removed from its endogenous position argues in favor of the timing changes being a consequence of centromere proximity. Alternatively, it is conceivable that there is an uncharacterized sequence element, distinct from centromeric sequence, residing in pericentric DNA that is promoting early activation of nearby origins. The existence of such an element is formally possible as a cryptic sequence residing at the 3′ end of the *URA3* gene was shown to advance the activation time of nearby origins on a plasmid and in an artificial chromosomal setting [25], [26]. We tested these possibilities as described below.

**Figure 4:**
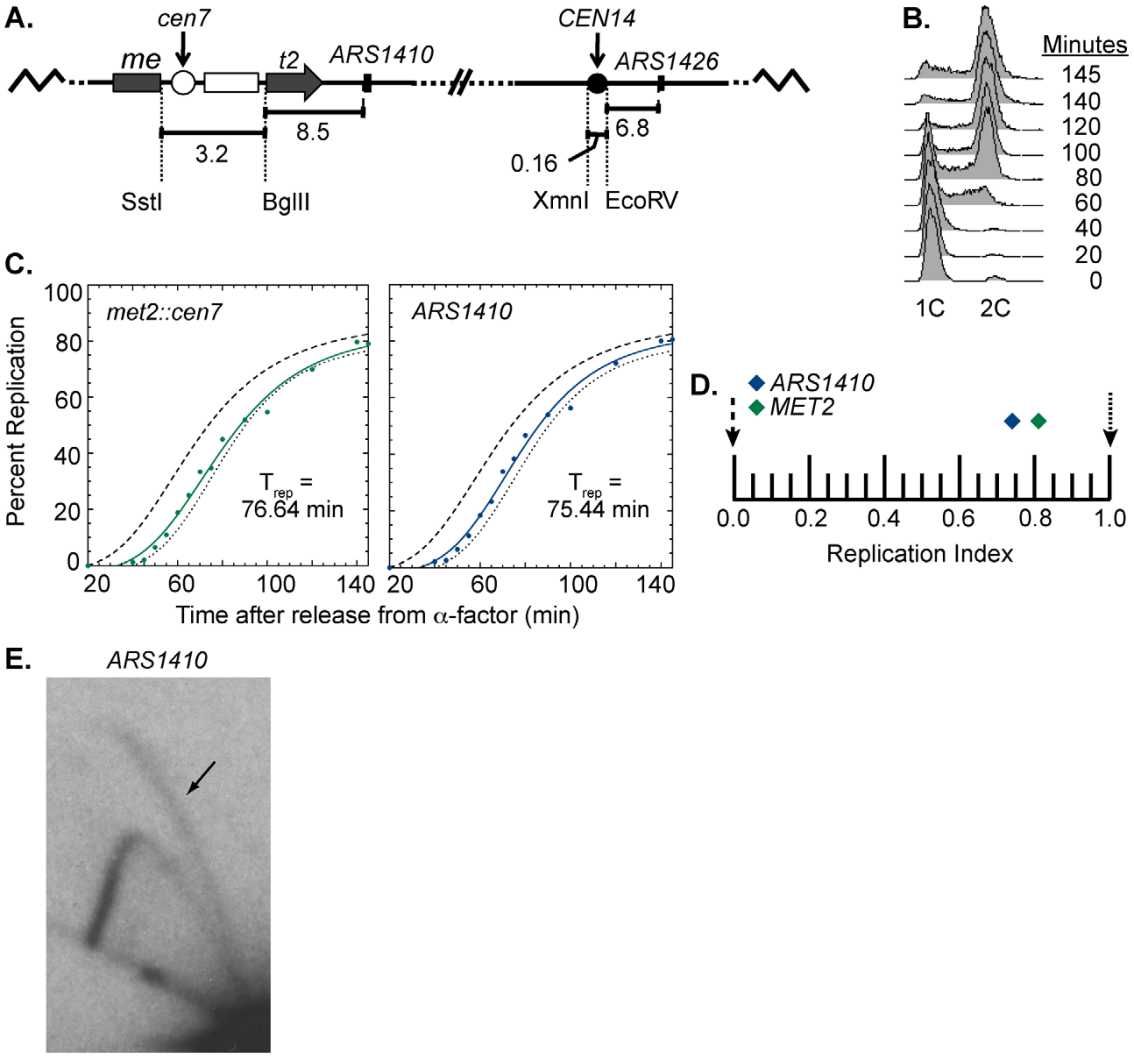
Replication time of point mutated centromere on chromosome XIV. (A) Cartoon depiction of chromosome XIV with a non-functional centromere (white circle) integrated at MET2. Chromosome XIV of WT cells was modified such that MET2 was disrupted with the same sequence used to disrupt MET2 in the rearranged strain (see Figure 1) except that the centromere was made inactive by mutating the essential CDEIII domain. This chromosome is maintained through its wild type centromere at the endogenous location (black circle). (B) Flow cytometry of cells with a non-functional centromere in the MET2 locus. Similar to the WT strain (see Figure 2B), cells from this cell line enter S-phase by 40 minutes and achieved 2C DNA content by 140 minutes. (C) Replication kinetic curves for met2::cen7 and ARS1410. As observed in the WT strain, the replication curves for met2::cen7 (green) and ARS1410 (blue) are positioned more closely to that of R11 (dotted line) than ARS306 (dashed line) (compare to Figure 2C). D) Replication indices for met2:cen7 (green diamond) and ARS1410 (blue diamond). RIs of ARS306 and R11 are indicated by black dashed and dotted arrows, respectively. met2:cen7 and ARS1410 had RIs of 0.81 and 0.74, respectively. (E) 2D gel analysis of ARS1410. Presence of a bubble arc (black arrow) for ARS1410 in the strain in which the non-functional centromere was integrated at MET2 (compare with Figure 1B) indicates that ARS1410 is a functional origin in this strain.

At the sequence level, *S. cerevisiae* centromeres are composed of three essential elements: CDEI, CDEII, and CDEIII. CDEI and CDEIII are defined by essential consensus sequences whereas CDEII is a 78–86 bp AT-rich sequence that separates CDEI and CDEIII [1], [19], [20]. CDEIII has been found to be the element most important for centromere function as it is the binding site for essential inner kinetochore proteins, notably members of the CBF3 complex [1], [19], [30]. To ask if a functional centromere is required for early activation of nearby origins, we engineered a strain to have a non-functional centromere with a mutated CDEIII motif integrated at the *MET2* locus while the functional centromere remained in its endogenous position (Figure 4A). This strain was also subjected to flow cytometry (Figure 4B) and replication timing analysis as described above (Figure 4C). Unlike the dramatic replication timing change observed when we introduced a functional centromere, introducing the mutated centromere caused no replication timing change at *met2* and *ARS1410* (RI of 0.81 and 0.74, respectively; Figure 4D). Therefore, we conclude that centromere function is needed to effect a timing change on nearby origins. Furthermore, the late replication of this region was not due to inactivation of *ARS1410* through insertion of the mutated centromere as indicated by 2D gel analysis (Figure 4E). Together, these data demonstrate that functional centromeres actively advance the activation times of origins over a distance of 11.5 kb.

### Centromeres determine the replication time of their local genomic environment

Upon finding that centromeres advance the activation time of origins to a distance of at least 11.5 kb, we sought to determine how far the centromere's effect extends along the chromosome. Raghuraman et al. presented statistical evidence that the regions of chromosomes within 25 kb of centromeres replicated earlier than the genome average, raising the possibility that centromeres could influence origin activation time over this larger distance [6]. Interestingly, at least one early replicating origin can be found within 12.8 kb of every centromere [16], suggesting that centromeres may be able to influence the activation time of origins over at least this distance. A recent study using an S-phase cyclin mutant [7] showed that early replicating domains that include a centromere can be well over 100 kb, implying that the range over which centromeres can regulate origin activation time might be quite broad.

**Figure 5:**
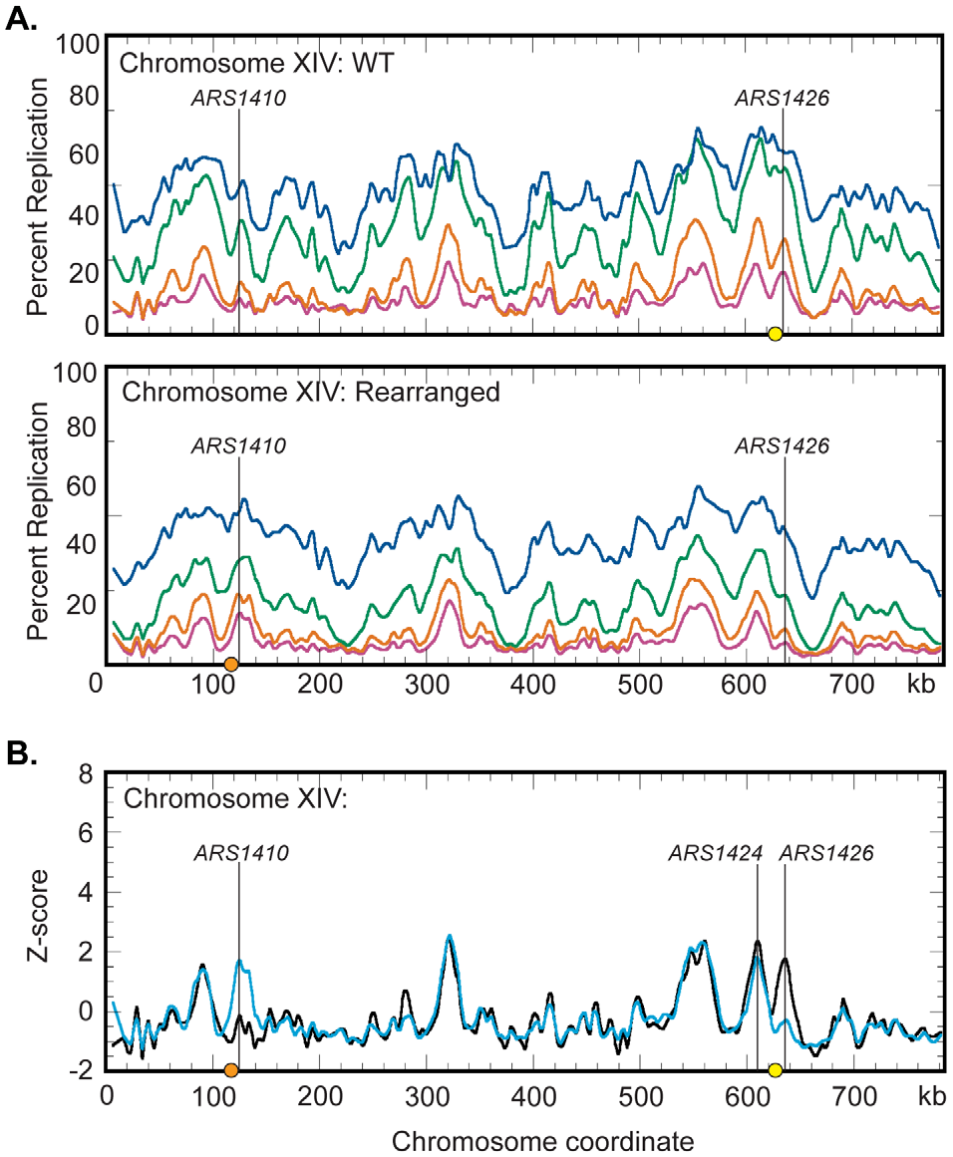
Replication dynamics for chromosome XIV in WT and rearranged strains. (A) Replication kinetic profiles of chromosome XIV in WT (top) and rearranged (bottom) strains. Percent replication was monitored across chromosome XIV at 40 (magenta), 45 (orange), 55 (green), and 65 (blue) minutes following release from alpha factor arrest. When the native centromere (yellow circle) is present near ARS1426, a prominent peak is seen in the 40 and 45 minute time samples. In this strain, the peak at ARS1410 is shallow in the 40 and 45 minute samples. When the centromere is repositioned (orange circle) near ARS1410 in the rearranged strain, both the time of appearance and the prominence of the peaks at ARS1410 and ARS1426 are inverted with respect to the WT strain. See Figures S2 and S3 and Datasets S1 and S2 for all chromosomes. (B) Z-score plots of chromosome XIV in WT (black) and rearranged (blue) strains. Replication kinetic profiles from the 40 minute sample were normalized by converting percent replication values to Z-score values (see Materials and Methods). Genomic loci corresponding to ARS1410 and ARS1426 show significant differences in Z-scores. ARS1424 is the next closest active origin to the endogenous centromere residing ∼19 kb to the left. See Figures S4, S5, S6 and Datasets S3 and S4 for all chromosomes and the 45- and 65-minute samples.

To determine the range over which a centromere can influence replication time we performed a genome wide analysis of replication in the WT and rearranged strains. Cells were grown in dense medium (see Materials and Methods) and timed samples were collected following release into S-phase in light medium. To obtain a genome-wide view, the HH and HL DNAs from each timed sample were labeled with different fluorophores, cohybridized to microarray slides, and replication profiles were generated (Figure 5A, Figures S2 and S3). Peak locations in the profile correspond to the locations of origins while the timed sample in which the peaks first appear gives an indication of the time at which the corresponding origins become active during S-phase [6].

Replication profiles generated for the WT strain (Figure S2) were consistent with previous studies [8]. Chromosome XIV displayed a strong peak at *ARS1426* at the earliest time (40 minutes) in the time course and a less well-defined peak at *ARS1410* (Figure 5A). Conversely, replication profiles in the rearranged strain displayed a shallow and late appearing peak at *ARS1426* while the peak at *ARS1410* appeared strong and early (Figure 5A and Figure S3) confirming the observations made by slot blot analysis of individual restriction fragments. To directly compare replication profiles from the two strains, the percent replication values from the 40, 45, and 65 minute samples were normalized by conversion to Z-scores (see Materials and Methods) and superimposed on the same axes (Figure 5B; Figures S4, S5, and S6).

Comparison of the Z-scores on chromosome XIV (Figure 5B) indicates that centromeres have a drastic influence over the activation times of their closest origins, suggesting that centromeres, mechanistically, operate locally. Z-score profiles for WT and rearranged chromosomes display a prominent early appearing peak centered about 19 kb to the left of the endogenous centromere. *ARS1425* is a potential origin located between *ARS1424* and the endogenous centromere [31]. However, 2D gel analysis demonstrated that *ARS1424* is the origin likely responsible for this peak as *ARS1425* is not active in either strain (data not shown). Replication timing analysis of *ARS1424* by slot blot hybridization indicates that this origin is influenced by the centromere to a far lesser extent than *ARS1426* (WT RI = 0.11, Rearranged RI = 0.27). That this mild effect on *ARS1424* is not reflected in the Z-score overlays is likely due to the higher resolution of slot blot analysis compared to microarray analysis.

**Figure 6:**
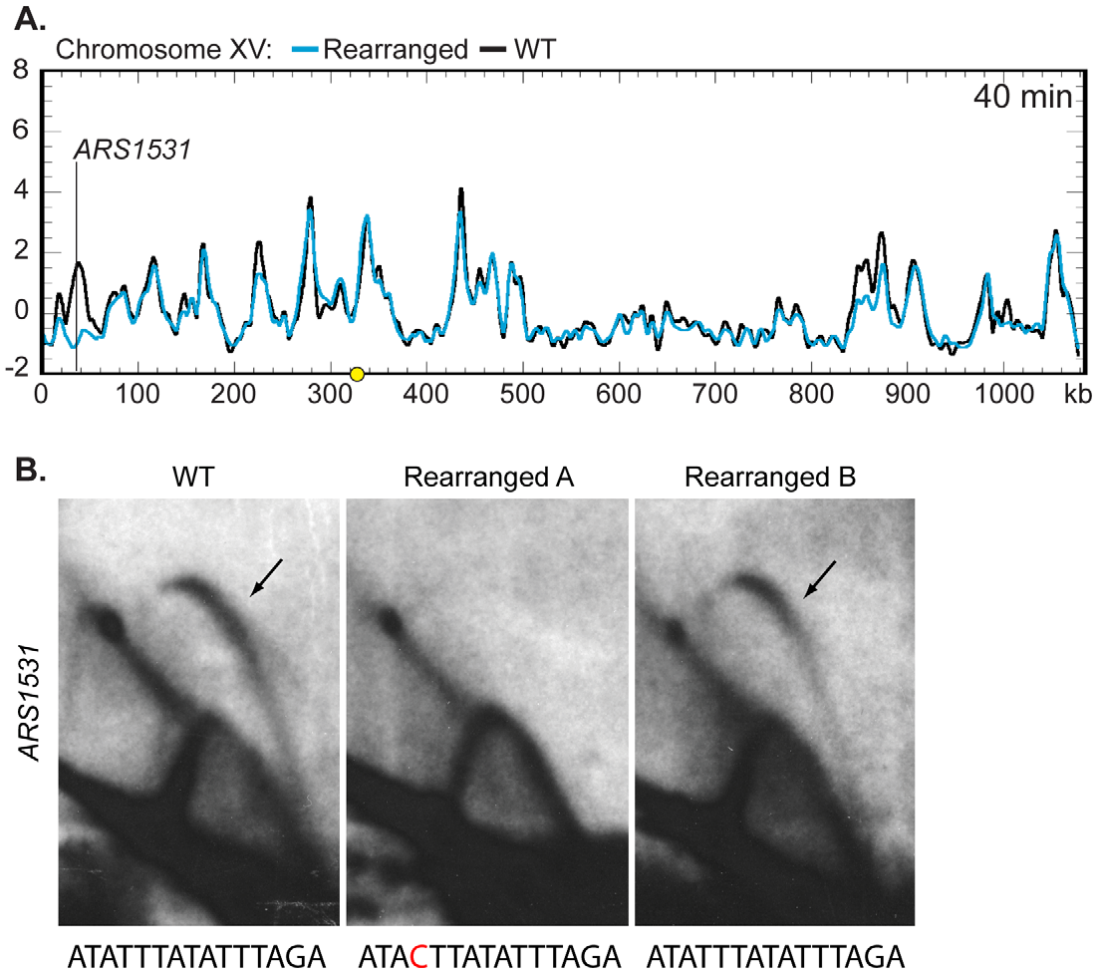
Z-score and 2D gel analysis of ARS1531. (A) Z-score plot of chromosome XV in WT (black) and rearranged (blue) strains. ARS1531 displayed a difference of Z-score values at least as large as that seen for ARS1410 and ARS1426 (see Figure 5B). See Figure S4 for all chromosomes. (B) 2D gel analysis of ARS1531 in the WT (left), the rearranged strain used in microarray analysis (middle), and the rearranged strain used for prior slot blot analysis (right). DNA from all three strains was digested with NcoI and BglII to give a 3.18 kb fragment harboring ARS1531 and then subjected to 2D gel analysis. The presence of a bubble arc in the WT (black arrow) indicates that ARS1531 is a functional origin in this strain. The presence of a bubble arc in one of the two rearranged strains confirms that the absence of origin activity in rearranged A (used in microarray analysis) is not due to relocation of the centromere on chromosome XIV. Below each 2D gel image is the sequence for the WT or mutant (red) ACS.

We then asked if the moved centromere influenced the activation times of origins located on other chromosomes. The replication kinetics of *ARS1*, a well-characterized origin on chromosome IV, were examined by slot blot hybridization and found to be similar between the two strains (WT RI = 0.68, Rearranged RI = 0.66) indicating that the activation time of *ARS1* is not influenced by centromere position on chromosome XIV. The remaining profiles were examined by Z-score comparisons looking for possible *trans* effects of centromere position. The profiles were strikingly similar (Figures S4, S5, and S6). Only one other location, corresponding to *ARS1531* on chromosome XV, showed a timing difference as drastic as that seen for *ARS1410* and *ARS1426* (Figure 6A; Figures S4, S5, S6). A prominent peak of high percent replication corresponding to *ARS1531* was present in the WT strain while no peak was observed in the rearranged strain. However, further examination using 2D gel electrophoresis revealed that although *ARS1531* is not an active origin in the isolate of the rearranged strain used for microarray analysis it is active in the WT strain as well as in the rearranged independent segregant used in the slot blot analysis of timing (Figure 6B; see Figure S1 for kinetic data for the independent segregant). These data suggest that the apparent timing change at this chromosome location is likely to be a consequence of a polymorphism affecting the ability of *ARS1531* to function as an origin rather than from any long-range effect of the centromere. Sequencing of *ARS1531* revealed that this origin in strain YTP16 contains a mutation in the essential ARS consensus sequence (ACS) [31] converting it from ATATTTATATTTAGA to ATACTTATATTTAGA (Figure 6B). A change of the T to a C at this position has been shown to entirely abolish origin activity in *ARS307* [32]. *ARS1531* does not contain this substitution in either YTP12 or YTP15. To confirm that this basepair change is responsible for the observed difference in origin activity, *ARS1531* was cloned from YTP12 and YTP16 and tested for its ability to produce transformants in YTP12, YTP16, and YTP19. The “T” version of *ARS1531* resulted in the formation of many robust colonies in the three strains tested whereas the “C” version of *ARS1531* did not (data not shown).

## Discussion

In this study we investigated the long-standing question of why centromeres replicate early in S-phase. We considered two possibilities: (1) that some component required for centromere function is also involved in early origin activation, or (2) that evolution has favored the migration of centromeres to early replicating regions. It has been hypothesized that early origin initiation in *S. cerevisiae* is the default state for origin timing [24], suggesting a more passive mechanism for early centromere replication. However, the observation that early centromere replication is conserved [9]–[11] in conjunction with the identification of a DNA element that is not associated with centromeres but capable of advancing origin activation time [25], [26] indicates that establishment of early origin activation time is more complex than previously thought. Consistent with this idea, a recent study shows that the centromeres of *C. albicans* can alter the replication times of the loci in which they reside by allowing the formation of a *de novo* early firing origin [11]. Because the neocentromere in *C. albicans* creates a new origin, it is unclear whether *C. albicans* centromeres also directly influence origin activation time. These results raised the question of whether the relocation of a centromere would have a similar effect in organisms with point centromeres. In this study, we took advantage of the well-characterized centromeres and origins in *S. cerevisiae* to effectively separate the centromere from origin function and address these questions.

We show that centromeres in *S. cerevisiae* are capable of advancing the replication time of genomic regions in which they reside by inducing early activation of their adjacent origins at distances of 11.5 and 6.8 kb (compare *ARS1410* and *ARS1426* in WT and rearranged strains, Figure 2C and 2D). We also show that early activation of *ARS1410* and *ARS1426* depends on centromere function. We find that centromere-mediated early origin activation requires an intact CDEIII region, suggesting that early origin activation is dependent on at least some portion of the DNA-protein complex normally formed at the centromere. Thus, centromeres and at least a subset of the kinetochore proteins they assemble participate as *cis*-acting regulatory elements of origin firing time. Furthermore, our 2D gel results indicate that centromeres do not affect origin efficiency, suggesting that the mechanisms responsible for centromere-mediated early origin activation are distinct from those that determine efficiency.

*S. cerevisiae* centromeres are known to reside in large early replicating portions of the genome spanning as much as 100 kb and containing multiple origins [5]–[8]. In light of the finding that centromeres regulate the activation times of their closest origins, we were interested in determining over what distance centromeres exert their effect. Our genome-wide replication timing analysis indicates that the centromere's influence on origin activation time is severely diminished at a distance of ∼19 kb (Figure 5B and 5C) indicating that not all early replicating origins are under the centromere's influence. This result implies that there are at least two distinct mechanisms by which origins can fire early. In contrast to what has been observed in *C. albicans*, we see no evidence that the centromeres of *S. cerevisiae* create new origins.

The genome is not randomly organized within the nucleus but particular genomic regions co-localize or cluster into functional foci during processes such as DNA replication [33], [34]. In particular, centromeres in *S. cerevisiae* cluster throughout the cell cycle [35]. Therefore, it is plausible that centromeres, their neighboring origins, as well as other portions of the genome that interact with them, are clustered in G1 phase when timing decisions are made. This clustering could provide a way for centromeres to mediate replication time through a *trans*-acting mechanism. To determine if the mechanism responsible for centromere mediated early origin activation is capable of acting in *trans*, we examined the genome wide replication timing data for other timing changes occurring in the genome as a result of the repositioned centromere on chromosome XIV. We looked for differences in the Z-score profiles between matched S-phase samples, demanding that they persist over the course of S-phase (See Figures S4, S5, S6) to be considered significant.

Upon inspection, only one other location centered on *ARS1531* displayed a timing difference of at least the same magnitude as observed for *ARS1410* or *ARS1426* (compare Figure 6A; Figures S4, S5, S6). 2D gel analysis and sequencing indicate that this timing difference is not a result of relocating the centromere position on chromosome XIV and that it is likely due to a polymorphism affecting the ability of *ARS1531* to function in this particular isolate. Other than these three locations, the replication profiles are remarkably similar, suggesting that the mechanisms by which centromeres influence origin activation time are restricted to relatively limited adjacent regions. However, it is possible that centromeres contribute to smaller timing differences observed on other chromosomes such as that observed on chromosome XV at ∼850 kb (Figure 6A; Figures S4, S5, S6). Because the timing change at this location was only present in two of the three Z-scored samples, we did not consider it to be a significant timing difference.

**Figure 7:**
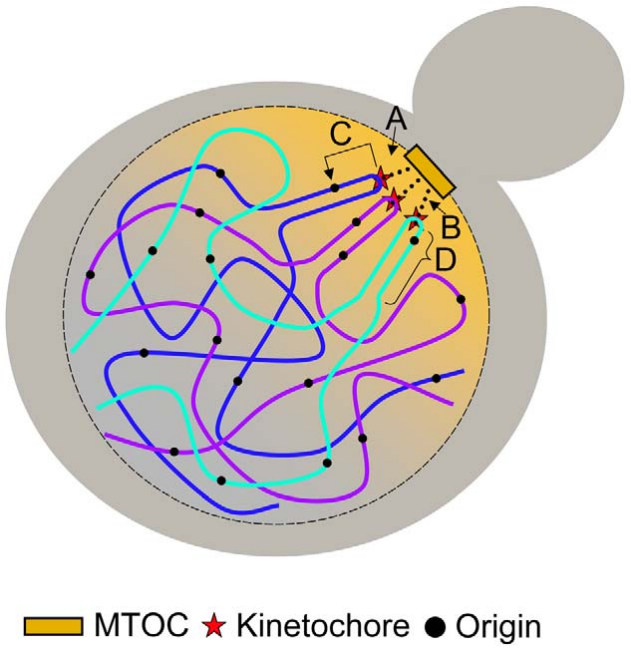
Models for centromere-mediated early origin activation. (A) Kinetochore/microtubule interaction orients the centromere and pericentric DNA near the microtubule organizing center (MTOC) where there is an enrichment of replication initiation factors. (B) Tension exerted by the kinetochore/microtubule interaction induces an altered chromatin structure of pericentric DNA that provides accessibility of embedded origins to initiation factors. (C) Kinetochore proteins interact directly (or indirectly) with origin initiation factors recruiting them to nearby origins. (D) The organization of pericentric DNA into the C-loop orients origins within the C-loop to the periphery of the chromatin mass increasing their accessibility to initiation factors.

Here we show direct evidence of a molecular link between the establishment of the kinetochore and replication initiation machinery. Although the mechanism of centromere-mediated early origin activation is unknown, we show that such a mechanism is dependent on at least some of the protein components associated with the kinetochore that require an intact CDEIII region. We also show that the mechanism is capable of affecting initiation time of origins that reside up to ∼19 kb away from the centromere. We propose four possible models, which are not mutually exclusive (Figure 7): (1) The nuclear environment near the microtubule organizing center (MTOC) is particularly enriched in replication initiation factors; (2) The tension exerted by the microtubule is translated along the nearby DNA, altering its chromatin structure, thereby influencing the accessibility of the imbedded origins to initiation factors; (3) Proteins within the kinetochore directly (or indirectly) interact with initiation factors, recruiting them to nearby origins; and (4) The C-loop architecture of the pericentric chromatin (see below) ensures the origins within the C-loop will be at the periphery of the chromatin mass and are therefore more exposed to initiation factors. Studies examining the concentration of replication initiation proteins near MTOCs have not been conducted; however, nuclear pore complexes are enriched near MTOCs in *S. cerevisiae* [36], [37], the significance of which is unknown. While it is tempting to invoke localization to the nuclear periphery in the vicinity of MTOCs as a potential link between replication timing and centromeres, there is as yet no clear causal connection between nuclear localization and replication timing. Late origins tend to dwell at the nuclear periphery while early origins tend to be internal to the nucleus [38]. However, tethering an early origin to the nuclear periphery is not sufficient to alter its replication time [39] and conversely, delocalizing telomeres from the nuclear periphery does not advance their replication time [40]. Although experiments that test for protein-protein, protein-DNA, and DNA-DNA interactions of kinetochore/centromeric components have been reported, we are not aware of any experiments that bear directly on the second model (tension-mediated promotion of early origin activation).

Kinetochores are multiprotein complexes composed of over 60 proteins [19], [41]. Some of these proteins have been shown to interact both genetically and physically with proteins involved in regulating DNA replication [42]–[44]. However, as we are interested in molecular components that specify origin initiation time and these components have yet to be identified, it is not straightforward to determine which interacting protein candidates should be singled out for further study. A systematic way to parse through the various kinetochore components would be to determine if members of the inner-, mid-, or outer-kinetochore complexes are required for early origin initiation through the use of temperature sensitive alleles. Though this method poses it own set of challenges, it may prove to be fruitful in uncovering some of the mechanisms by which centromeres regulate origin activation time.

In vitro studies of protein-DNA interactions have shown that the CBF3 complex (composed of inner kinetochore DNA binding proteins) induces a 55° bend in centromeric DNA [45]. In concordance with this finding, more recent work from the Bloom lab has shown that pericentric DNA adopts a particular tertiary structure coined the C-loop [46]. The C-loop is characterized by an intramolecular loop centered on the centromere resembling a hairpin of duplex DNA. C-loop formation is dependent on a subset of inner kinetochore proteins that physically bind centromere DNA, namely those that bind the CDEIII region [46], [47]. Cohesin, which is enriched in pericentromeric regions [48]–[50], is thought to stabilize the C-loop following centromere replication [46]. Additonally, this study reports that the C-loop is present in G1-phase during alpha factor arrest, suggesting that neither the presence of a sister chromatid nor pericentric cohesin is essential for the maintenance of the structure. Interestingly, the intramolecular interaction of the C-loop is reported to extend more than ∼11.5 kb and less than 25 kb from the centromere, a distance similar to the findings reported in this study over which centromeres can regulate origin activation time. Anderson et al. hypothesized that proteins involved in the C-loop formation provide an essential geometry required for centromere position and accessibility for microtubule binding [47]. In light of these data, it is tempting to hypothesize that this geometry provides a favorable environment for early origin initiation by increasing the accessibility of nearby origins to replication initiation machinery.

Although the DNA binding components of the inner kinetochore are not well conserved, in human cells a DNA binding component of the inner kinetochore, CENP-B, has been shown to induce a ∼59° bend in its target sequence [51], suggesting that regional centromeres also adopt a particular configuration. Therefore, this model can also explain how centromeres can vary greatly at the sequence level yet still affect timing of their associated origins.

## Materials and Methods

### Yeast strains

Strain and vector genotypes can be found in Table S1. Primer sequences can be found in Table S2. All *S. cerevisiae* strains used in this study were derived from a cross of CH1870 obtained from [27] and S288C. CH1870 is a mating type alpha strain derived from the S288C background. The *MET2* locus of this strain (located on chromosome XIV) was disrupted by an insertion of centromere VII sequence and *LEU2*. The endogenous centromere on chromosome XIV of CH1870 was also replaced with a *URA3* selectable marker [27].

The *ura3-52* allele in YTP13 (derived from a CH1870 to S288C cross) was restored to *URA3* by gene replacement, resulting in YTP15 (WT strain in this study, Table S1). Restoration was confirmed by Southern blot analysis.

Independent segregants of the rearranged strains (YTP12 and YTP16) were obtained from separate tetrads from a CH1870 to S288C cross as met−, leu+, and ura+ progeny. Three different PCR reactions using primer pairs 71∶72, 72∶88, and 71∶126 were used to confirm that these strains contained the desired inserted sequence at the *MET2* locus (data not shown). The desired insertion was further confirmed by Southern blot analysis on genomic DNA that was digested with XbaI (data not shown).

### Mutant centromere at the met2 locus

YTP19 was constructed to have the same *met2::CEN7.LEU2* cassette as YTP12 and YTP16 save for a 3 bp mutation in CDEIII. The mutation of CDEIII in the centromere sequence integrated at *MET2* was made non-functional through site directed mutagenesis [52]. This strain was constructed as follows: Two halves of *met2*::*CEN7.LEU2* were PCR amplified from YTP12 using primer pairs 71∶133, and 72∶134. Primer pair 71∶133 was used to amplify the 5′ end of *met2* with primer 71 hybridizing upstream of the BsmI restriction site located in *met2* and primer 133 hybridizing downstream of the EcoRV restriction site located in the *LEU2* gene. Primer pair 72∶134 was used to amplify the 3′ end of *met2* with primer 72 hybridizing downstream of *met2* and primer 134 hybridizing upstream of the EcoRV restriction site located in the *LEU2* gene. The PCR products were sequentially digested with either BsmI or AatII followed by an EcoRV digest. The two fragments were then inserted into a pUC18 vector that contained KanMX and *ARS228* through a tri-molecular ligation reaction, creating plasmid pTP18.

The centromere on plasmid pTP18 was made non-functional, creating pTP19, through site directed mutagenesis using primers 147 and 148 [52]. These primers were used to mutate the Centromere DNA Element III (CDEIII) from TCCGAA to TCTAGA, introducing an XbaI site into the sequence. The most important bases for centromere function are the middle CG in TCCGAA [1], [45]. The mutated centromere sequence on pTP19 was Sanger sequenced to ensure that only CGA had been changed. The lack of centromere activity in pTP19 was confirmed by a plasmid stability assay in which serially diluted cells were spotted to selective medium over 24 generations (data not shown).

The 4025 bp AatII/BsmI fragment of plasmid pTP19 containing *met2*::*cen7*.*LEU2* was transformed into YTP15 [53], [54]. Transformants were selected on synthetic dropout (SD) medium plates lacking leucine. Cells were then replica-plated to SD medium lacking methionine. Colonies that were prototrophic for leucine and auxotrophic for methionine were further screened similar to the rearranged strains mentioned earlier using PCR primer pairs 71∶72 and 72∶88 and through Southern blot analysis on XbaI digested genomic DNA.

### Two-dimensional agarose gel electrophoresis

Origin activity was analyzed by standard 2D agarose gel electrophoresis techniques performed on total genomic DNA obtained from either asynchronous or synchronous S phase cells [55]–[57]. Asynchronous samples were used for 2D gels conducted on *ARS1426*, *ARS1531*, and *met2::CEN7*. Synchronized samples were used for 2D gels conducted on *ARS1410* and *MET2*. For asynchronous samples, cells were collected in early log phase. For synchronized samples, cells were arrested in G1-phase with alpha factor (final concentration 3 µM) and released synchronously into S-phase by the addition of pronase (0.15 mg/mL). Cells were then collected every 2 minutes and pooled. Genomic DNA was harvested similar to asynchronous samples. In the first dimension, DNA was separated in 0.4% agarose for 18–20 hours at 1 V/cm. The second dimension was run for 3–3.5 hours in 1.1% agarose containing Ethidium Bromide (0.3 µg/mL) at ∼5–6 V/cm at 4°C.

### Flow cytometry

Cells were harvested by mixing with 0.1% sodium azide in 0.2 M EDTA and then fixed with 70% ethanol. Flow cytometry was performed as previously described [58] upon staining cells with Sytox Green (Molecular Probes, Eugene, OR). The data were analyzed with CellQuest software (Becton-Dickinson, Franklin Lakes, NJ).

### Density transfer experiments

Density transfer experiments were performed as described in [4] with slight modifications in cell synchronization and sample preparation for microarray analysis [6]. Cells were grown overnight at 23°C in a 5 mL culture of dense medium containing ^13^C-glucose at 0.1% (w/v) and ^15^N-ammonium sulfate at 0.01% (w/v). Cells were then diluted into a larger vessel in dense medium and allowed to reach an optical density of 0.16 (∼2×10^6^ cells/mL). Cells were arrested in G1 by incubating with 3 µM alpha factor until at least 95% of cells were unbudded based on microscopic analysis. Cells were then filtered and transferred to medium containing ^12^C-glucose (2%), ^14^N-ammonium sulfate (0.5%), and alpha factor. Cells were then synchronously released from the G1 arrest through the addition of pronase at a concentration of 0.15 mg/mL. Samples were collected every 10 minutes. Cell samples were treated with a mixture of 0.1% odium azide and 0.2 M EDTA then pelleted and frozen at −20°C. Genomic DNA was extracted from pelleted cells, digested with EcoRI, and the DNA fragments were separated based on density by ultracentrifugation in cesium chloride gradients. Gradients were drip fractionated and slot blotted to nylon membrane where they were hybridized with probes of interest. Unreplicated DNA is HH in density while replicated DNA is HL in density.

### Replication kinetics and analysis

To construct replication kinetic curves, slot blots were hybridized to sequences of interest, and the percent replication in each timed sample was calculated as described in [4], [6], [8]. A detailed protocol can be found at http://fangman-brewer.genetics.washington.edu/density_transfer.html. The time at which the kinetic curves reach half maximal defines the time of replication (T_rep_) for each individual locus within the population of cycling cells. However, because not all G1 cells will enter or complete S-phase, T_rep_ for the probed regions in this study were calculated based on the plateau of the replication kinetic curve for a genomic probe consisting of EcoRI digested total genomic DNA from a strain lacking mitochondrial DNA.

To compare timing differences between strains, T_rep_ values were converted to replication indices in the following manner. First the T_rep_ values of two timing standards, *ARS306* (a known early replicating region) and R11 (a known late replicating region near *ARS501*), were obtained and assigned the values of 0 and 1, respectively. Next the T_rep_ values of regions of interest were assigned a replication index corresponding to the fraction of the *ARS306*-R11 interval elapsed at the time at which the T_rep_ for each locus was obtained. Replication index is calculated as (T_rep_(X)−T_rep_(*ARS306*))/( T_rep_(*ARS306*)−T_rep_(R11)) where X is the fragment of interest.

For microarray analysis, slot blots were hybridized with a genomic DNA probe to indentify fractions containing either HH or HL DNA. Once identified, the HH and HL fractions were separately pooled and differentially labeled with cyanine (Cy3 or Cy5) conjugated dUTP (Perkin Elmer) [8]. Timed samples were hybridized to high density Agilent yeast ChIP-to-chip 4×44 K arrays according to the manufacturer's recommendations. The algorithms for analyzing microarray data are described in [16]. An 18 kb window was used for overall smoothing.

To facilitate direct comparison of replication profiles, percent replication values obtained from microarray analysis were converted to Z-score values. Z-score values were calculated using the following formula: Z = (X−μ)/σ where X = the percent replication value for a given probe, μ = the genomic average percent replication for a given sample, and σ = the standard deviation of the distribution of X. The 55 minute samples were not used because the genomic percent replication values were not well matched between the two strains.

### Note added in proof

Two recent findings in yeast add interesting perspectives on the centromere effect on origin activation. First, the lack of forkhead proteins Fkh1 and Fkh2 results in delayed activation of a large number of normally early-firing origins, but centromere-proximal origins remain early-firing (Knott et al. 2012, Cell 148: 99–111). Second, while many origins show delayed activation in meiotic S phase compared to mitotic S, centromere-proximal origins do not show such a delay (Blitzblau et al., PLoS Genetics, in press). Both findings are consistent with the idea that some aspect of centromere function directly influences the activation of centromere-proximal origins through a mechanism that is independent of other, more global controls.

## Supporting Information

Dataset S1 Percent replication values for the wild type strain (shown in Figure 5 and Figure S2). Raw microarray signal intensity values for HH (unreplicated) and HL (replicated) DNA are included.
(XLS)

Dataset S2 Percent replication values for the rearranged strain (shown in Figure 5 and Figure S3). Raw microarray signal intensity values for HH (unreplicated) and HL (replicated) DNA are included.
(XLS)

Dataset S3 Z score values for the wild type strain (shown in Figure 5, Figure 6, and Figures S4, S5, S6).
(XLS)

Dataset S4 Z score values for the rearranged strain (shown in Figure 5, Figure 6, and Figures S4, S5, S6).
(XLS)

Figure S1 Replication kinetic data for an independent segregant of the rearranged strain. (A) Flow cytometry of independent segregant of the rearranged strain. The shift from 1C to 2C DNA content shows that cells entered S-phase at around 40 min and DNA synthesis was complete by 140 minutes (compare to Figure 2B). (B) Replication indices for met2 (green diamond), ARS1410 (blue diamond), and ARS1426 (magenta diamond) in the independent segregant of the rearranged strain were 0.26, 0.20, and 0.86, respectively. Timing standards, ARS306 and R11 are plotted as a black dashed arrow and a black dotted arrow, respectively (compare to Figure 2D).
(PDF)

Figure S2 Replication kinetic profiles for the WT strain. Microarray analysis was conducted on the 40 (magenta), 45 (orange), 55 (green), and 65 (blue) minute samples. Smoothed data are plotted for each of the 16 S. cerevisiae chromosomes.
(PDF)

Figure S3 Replication kinetic profiles for rearranged strain. Microarray analysis was conducted on the 40 (magenta), 45 (orange), 55 (green), and 65 (blue) minute samples. Smoothed data are plotted for each of the 16 S. cerevisiae chromosomes.
(PDF)

Figure S4 Comparison of WT and rearranged strain Z-score data for 40-minute samples. Replication kinetic data for the 40-minute samples in WT and rearranged cells were converted to Z scores and overlaid over the 16 S. cerevisiae chromosomes. WT data are plotted in black and rearranged data are plotted in blue. Endogenous and ectopic centromeres are depicted as yellow and orange circles, respectively.
(PDF)

Figure S5 Comparison of WT and rearranged stain Z-score data for 45-minute samples. Replication kinetic data for the 45-minute samples in WT and rearranged cells were converted to Z-scores and overlaid over the 16 S. cerevisiae chromosomes. WT data are plotted in black and rearranged data are plotted in blue. Endogenous and ectopic centromeres are depicted as yellow and orange circles, respectively.
(PDF)

Figure S6 Comparison of WT and rearranged strain Z-score data for 65-minute samples. Replication kinetic data for the 65-minute samples in WT and rearranged cells were converted to Z-scores and overlaid over the 16 S. cerevisiae chromosomes. WT data are plotted in black and rearranged data are plotted in blue. Endogenous and ectopic centromeres are depicted as yellow and orange circles, respectively.
(PDF)

Table S1 Strains and plasmids used in this study.
(DOC)

Table S2 Primers used during strain construction.
(DOC)
