# Supplementary figures and images for: Functional Centromeres Determine the Activation Time of Pericentric Origins of DNA Replication in Saccharomyces cerevisiae
Source: PLoS Genet. 2012 May 10;8(5):e1002677. doi: 10.1371/journal.pgen.1002677 (PMC3349730; doi:10.1371/journal.pgen.1002677)

Figure S1.

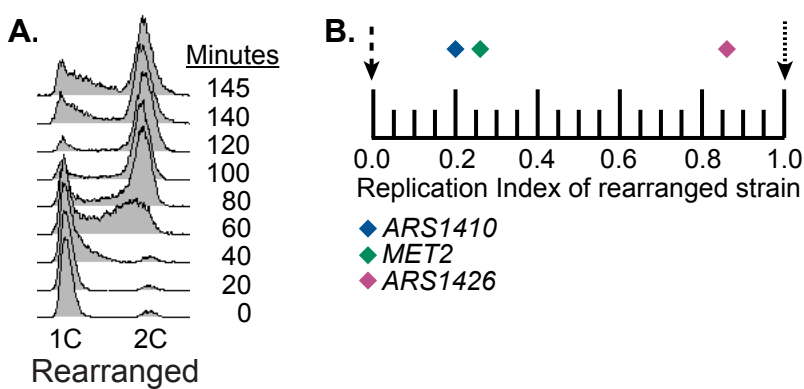

Supplement: Figure S1 — Replication kinetic data for an independent segregant of the rearranged strain. (A) Flow cytometry of independent segregant of the rearranged strain. The shift from 1C to 2C DNA content shows that cells entered S-phase at around 40 min and DNA synthesis was complete by 140 minutes (compare to Figure 2B). (B) Replication indices for met2 (green diamond), ARS1410 (blue diamond), and ARS1426 (magenta diamond) in the independent segregant of the rearranged strain were 0.26, 0.20, and 0.86, respectively. Timing standards, ARS306 and R11 are plotted as a black dashed arrow and a black dotted arrow, respectively (compare to Figure 2D). (PDF) [file pgen.1002677.s005.pdf]

**Figure S2. Replication profiles for wild type strain.**

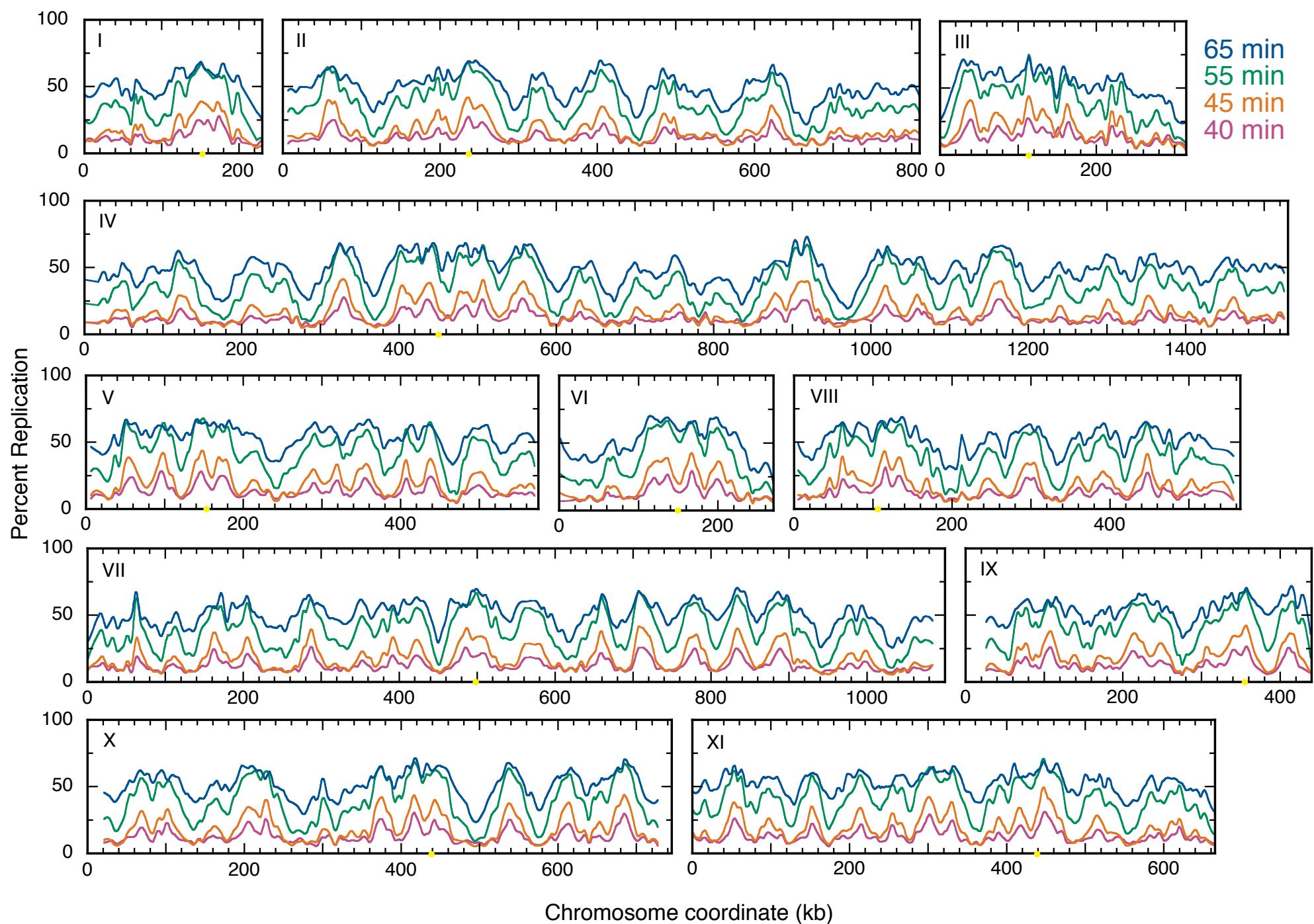

Figure S2 (cont'd)

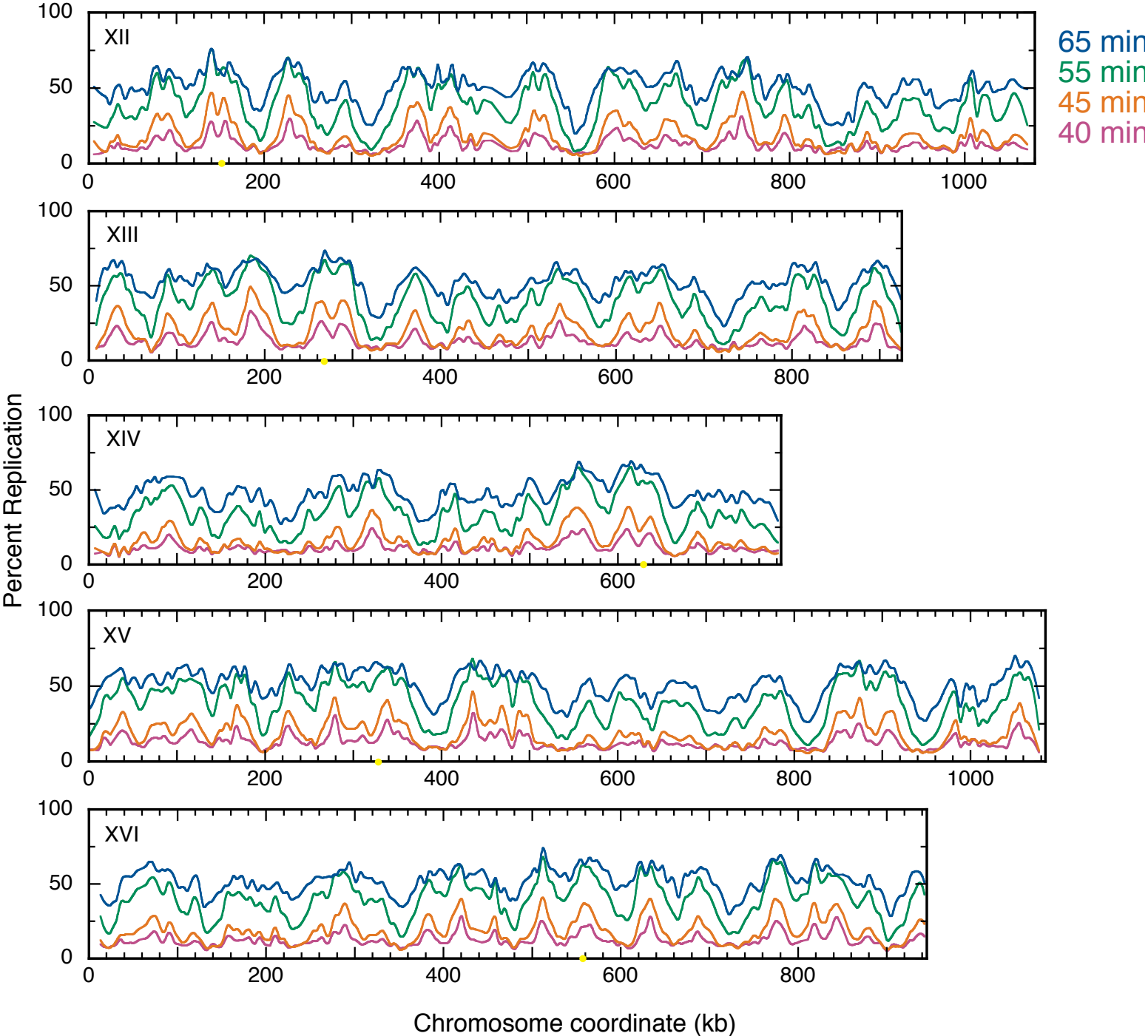

Supplement: Figure S2 — Replication kinetic profiles for the WT strain. Microarray analysis was conducted on the 40 (magenta), 45 (orange), 55 (green), and 65 (blue) minute samples. Smoothed data are plotted for each of the 16 S. cerevisiae chromosomes. (PDF) [file pgen.1002677.s006.pdf]

**Figure S3. Replication profiles for rearranged strain.**

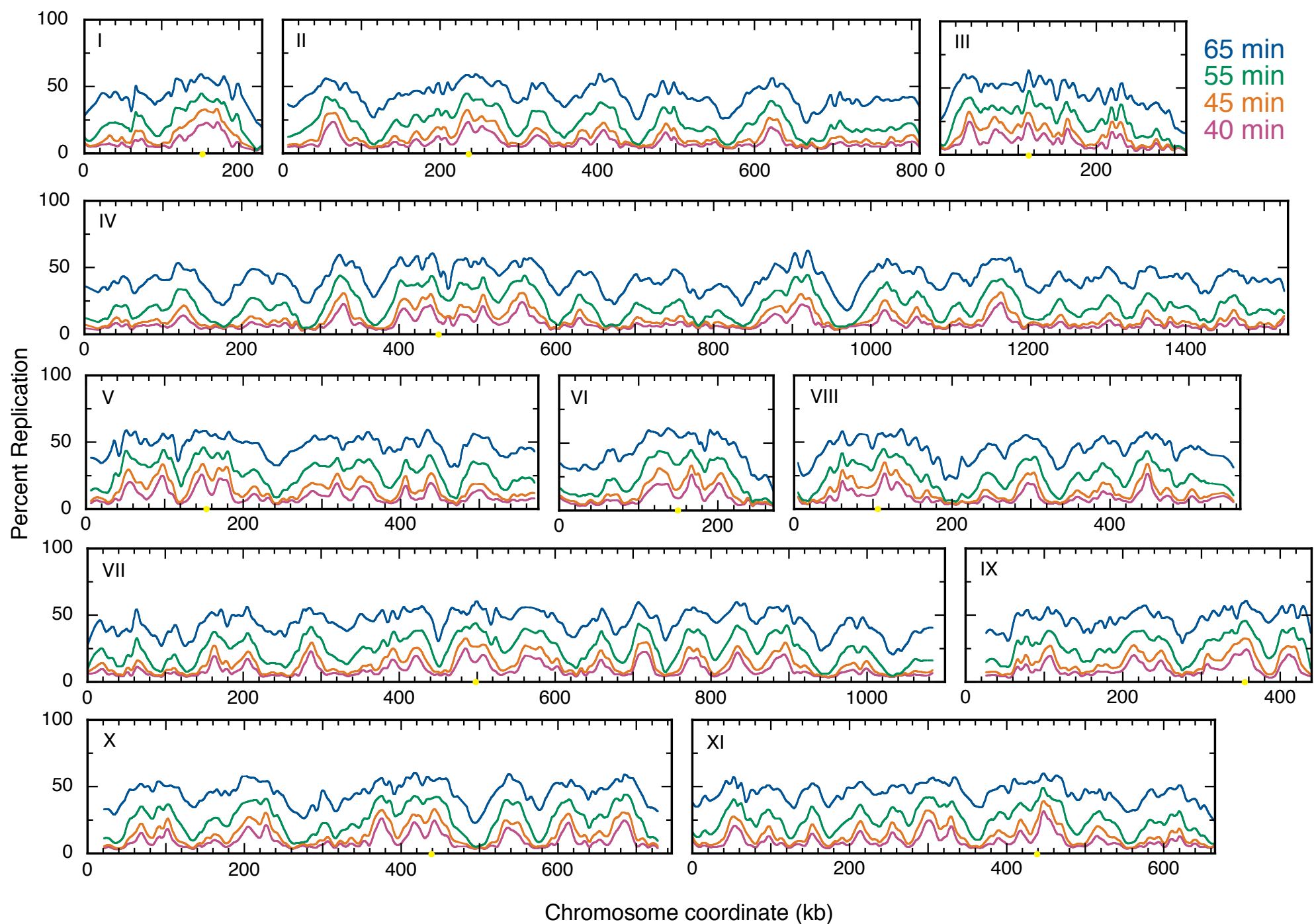

**Figure S3 (cont'd)**

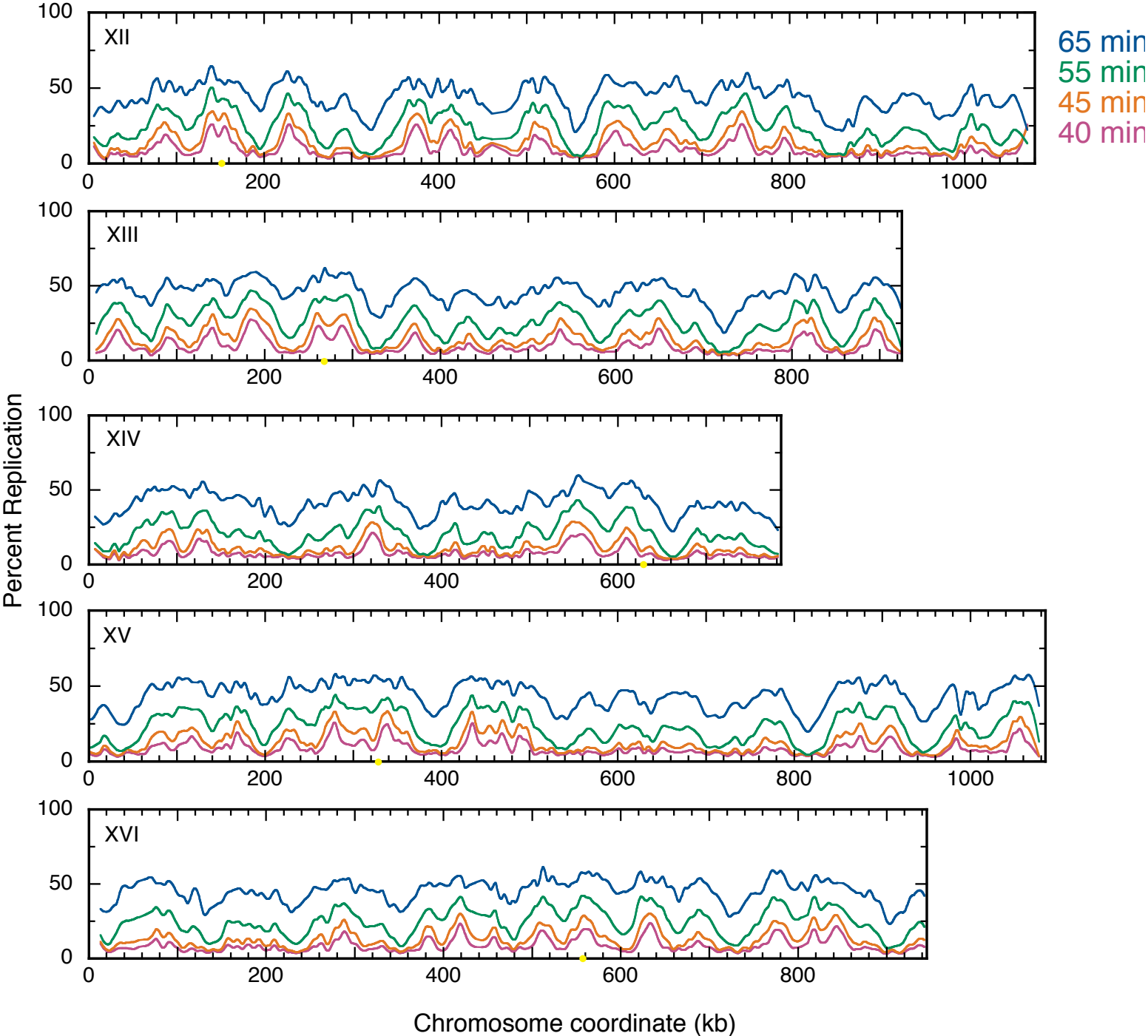

Supplement: Figure S3 — Replication kinetic profiles for rearranged strain. Microarray analysis was conducted on the 40 (magenta), 45 (orange), 55 (green), and 65 (blue) minute samples. Smoothed data are plotted for each of the 16 S. cerevisiae chromosomes. (PDF) [file pgen.1002677.s007.pdf]

Figure S4. WT vs. rearranged strain Z-scores, 40 min

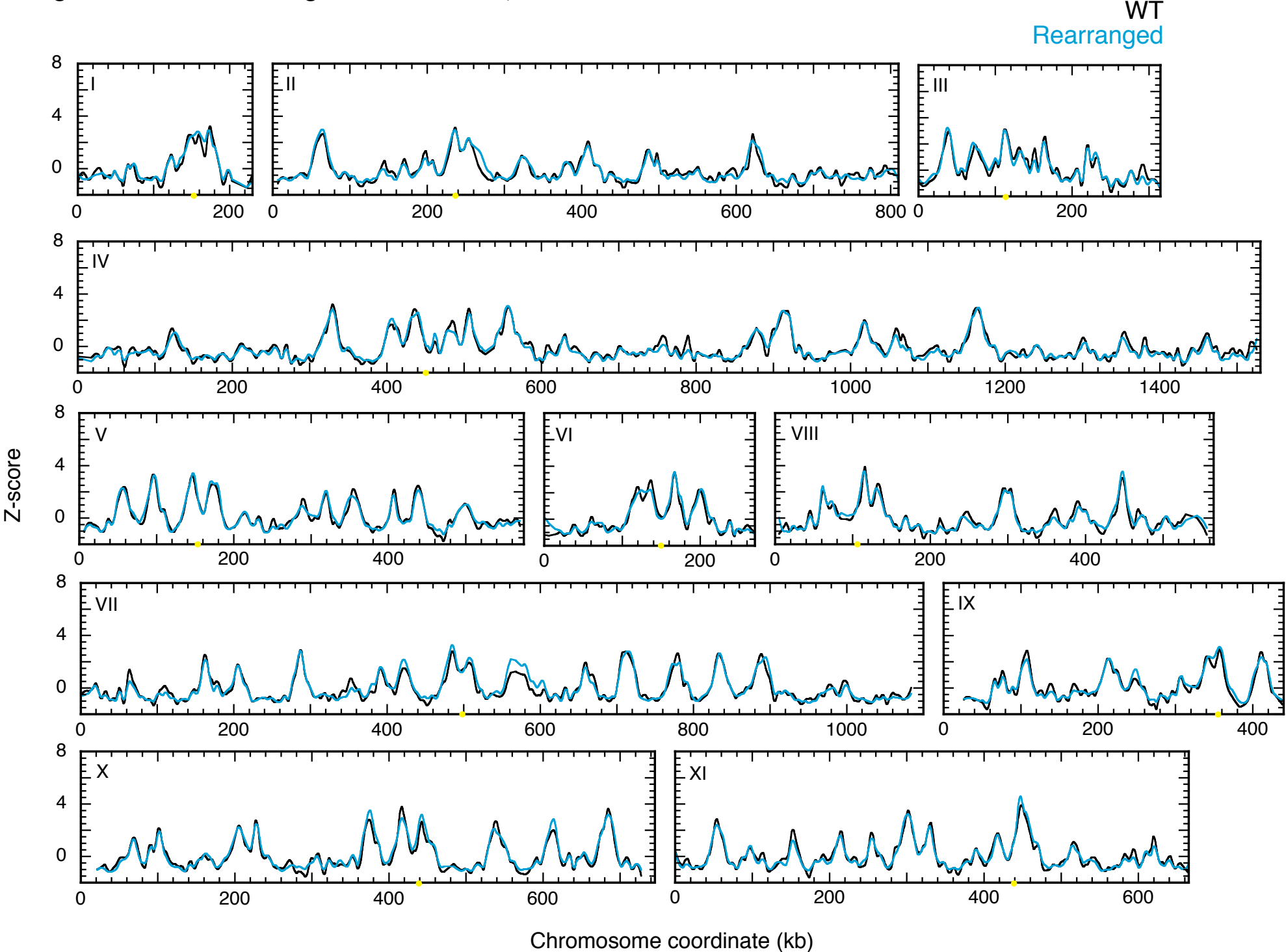

Figure S4 (cont'd)

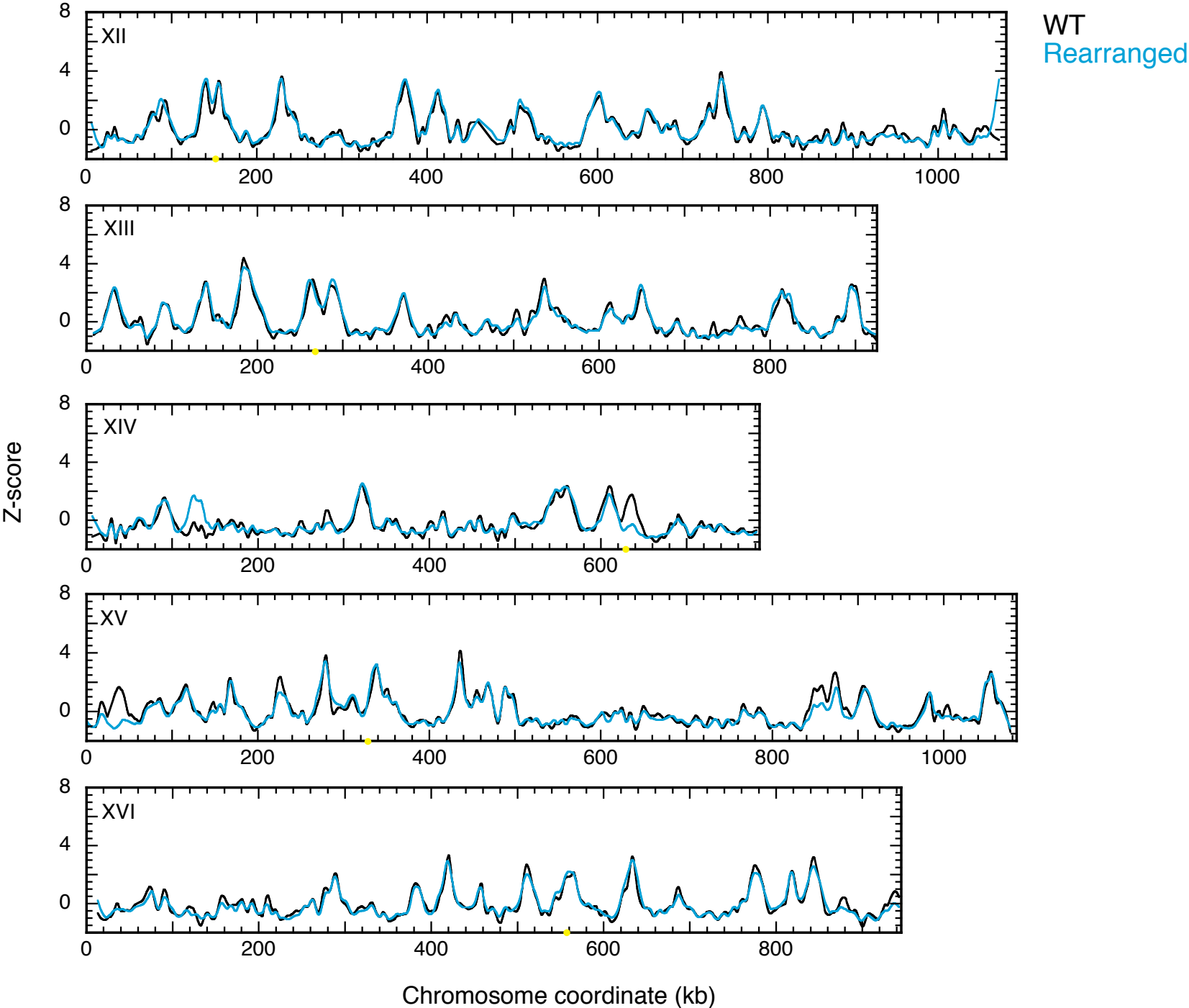

Supplement: Figure S4 — Comparison of WT and rearranged strain Z-score data for 40-minute samples. Replication kinetic data for the 40-minute samples in WT and rearranged cells were converted to Z scores and overlaid over the 16 S. cerevisiae chromosomes. WT data are plotted in black and rearranged data are plotted in blue. Endogenous and ectopic centromeres are depicted as yellow and orange circles, respectively. (PDF) [file pgen.1002677.s008.pdf]

Figure S5. WT vs. rearranged strain Z-scores, 45 min

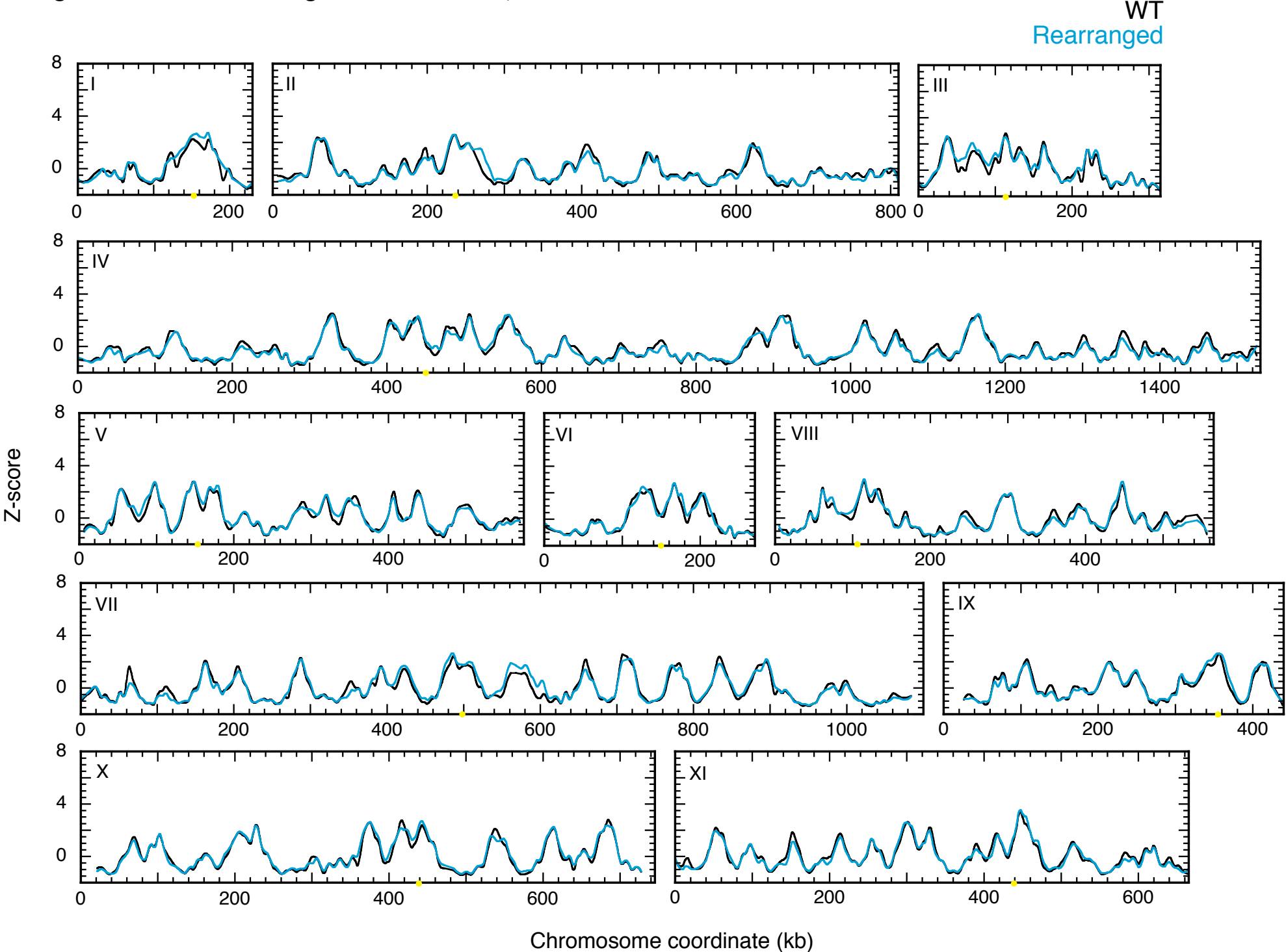

Figure S5 (cont'd)

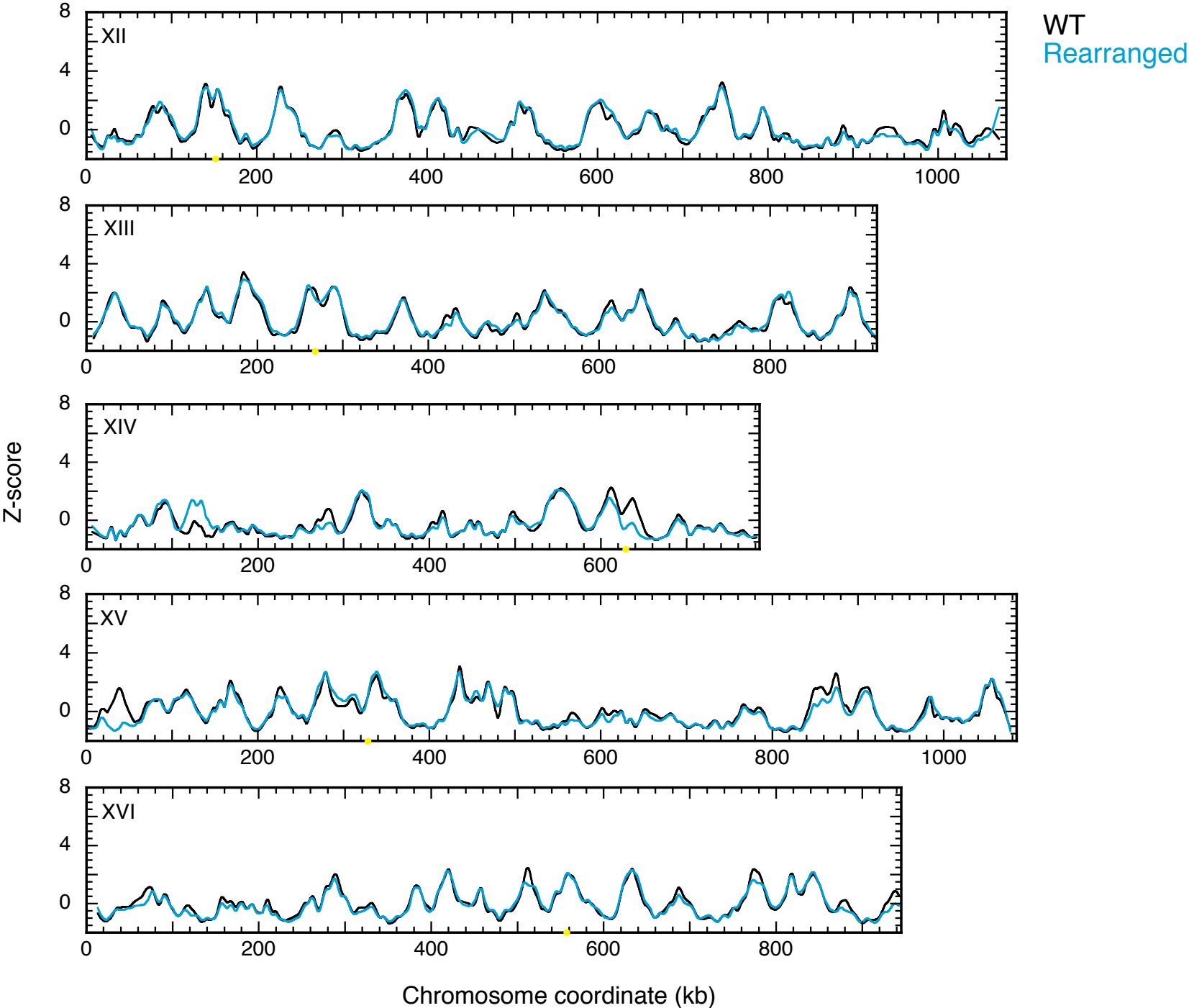

Supplement: Figure S5 — Comparison of WT and rearranged stain Z-score data for 45-minute samples. Replication kinetic data for the 45-minute samples in WT and rearranged cells were converted to Z-scores and overlaid over the 16 S. cerevisiae chromosomes. WT data are plotted in black and rearranged data are plotted in blue. Endogenous and ectopic centromeres are depicted as yellow and orange circles, respectively. (PDF) [file pgen.1002677.s009.pdf]

Figure S6. WT vs. rearranged strain Z-scores, 65 min

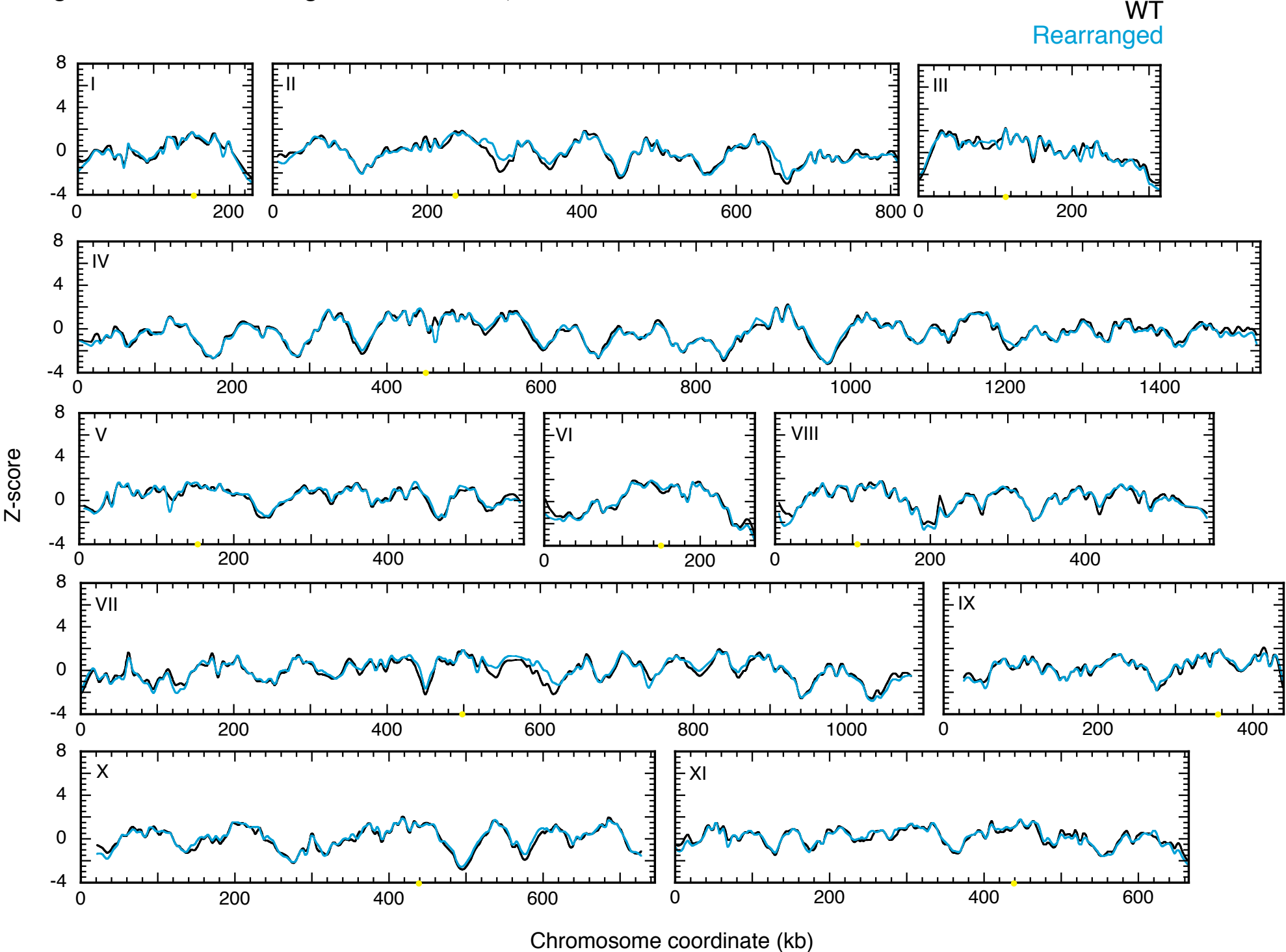

Figure S6 (cont'd)

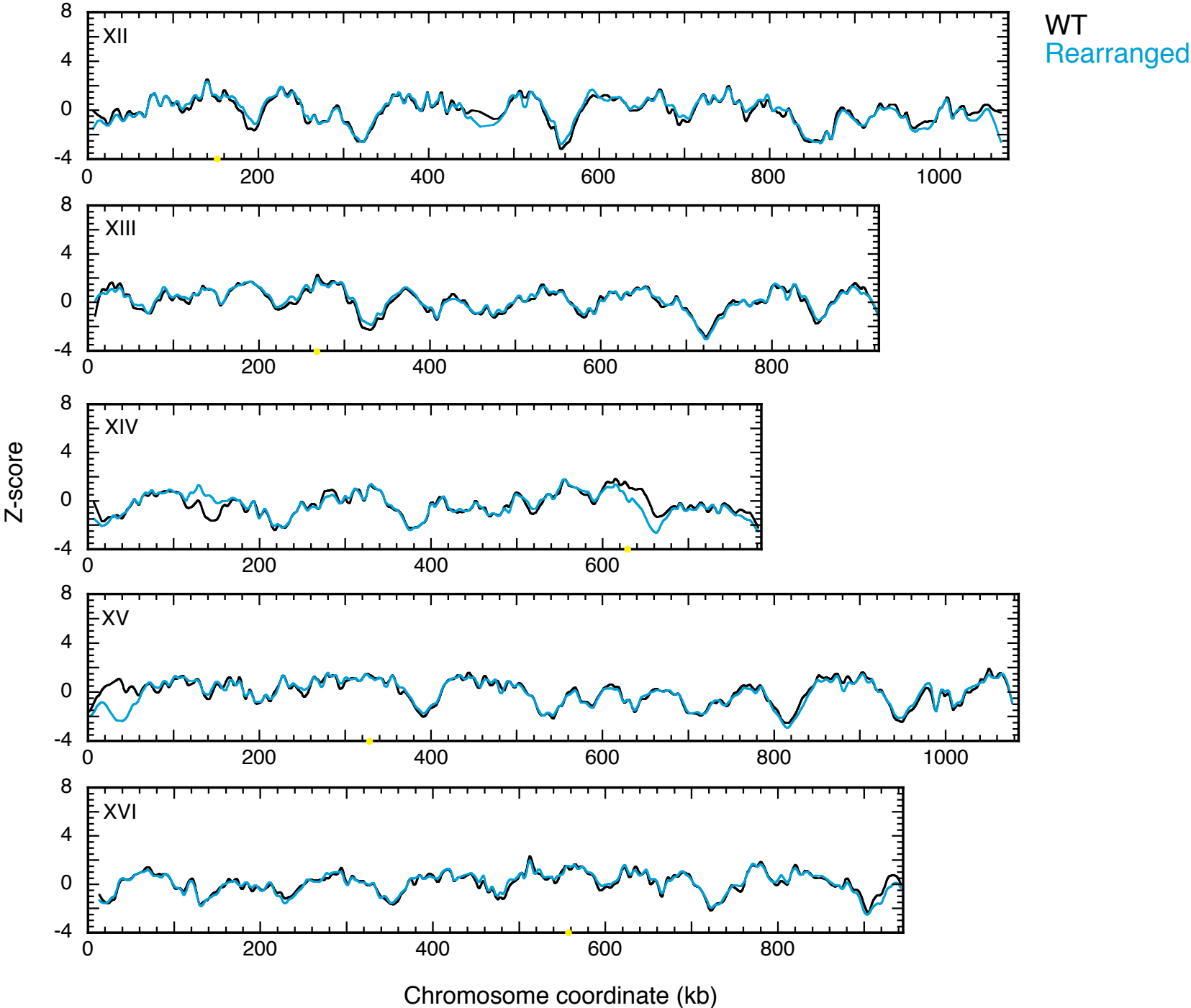

Supplement: Figure S6 — Comparison of WT and rearranged strain Z-score data for 65-minute samples. Replication kinetic data for the 65-minute samples in WT and rearranged cells were converted to Z-scores and overlaid over the 16 S. cerevisiae chromosomes. WT data are plotted in black and rearranged data are plotted in blue. Endogenous and ectopic centromeres are depicted as yellow and orange circles, respectively. (PDF) [file pgen.1002677.s010.pdf]
