# Supplementary material for: Functional Centromeres Determine the Activation Time of Pericentric Origins of DNA Replication in Saccharomyces cerevisiae
Source: PLoS Genet. 2012 May 10;8(5):e1002677. doi: 10.1371/journal.pgen.1002677 (PMC3349730; doi:10.1371/journal.pgen.1002677)
Supplement: Table S1 — Strains and plasmids used in this study. (DOC) [file pgen.1002677.s011.doc]

Supplemental Table S1. Yeast Strains and Plasmids

| Name | Genotype | Source |
| --- | --- | --- |
| CH1870 | *MATa*, *ura3-52*, *lys2-801am*, *leu2-3,112 met2*::*CEN7.LEU2*, *cen14*::*URA3* in an S288C background | (45) |
| S288C | *MATa*, *ura3-52*, *trp1*∆, *leu2-3,112*, *ade2-1*, *met-* (not *met2*) | (Byers lab) |
| YTP12 | *MATa,* *ura3-52*, *trp1*Δ, *leu2-3,112*, *lys2-801am*, *met2*::*CEN7.LEU2* *cen14*::*URA3* | This Study |
| YTP13 | *MATa*, *ura3-52*, *trp*Δ *leu2-3,112*, *lys2-801am* | This Study |
| YTP15 | *MATa*, *trp*∆, *leu2-3,112*, *lys2-801am* | This Study |
| YTP16 | *MATa*, *ura3-52*, *trp1Δ*, *leu2-3,112*, *lys2-801am*, *met2*::*CEN7.LEU2* *cen14*::*URA3* | This Study |
| YTP19 | *MATa*, *trp*Δ *leu2-3,112*, *lys2-801am*, *met2::cen7(cdeIII:XbaI).LEU2* | This Study |
|  |  |  |
| Plasmids | Genotype | Source |
| pUC18-KanMX-ARS228 | *Amp, KanMX, ScARS228* | Brewer lab |
| pTP18 | *Amp, ScARS228, met2::CEN7.LEU2* | This Study |
| pTP19 | *Amp, ScARS228, met2::cen7(cdeIII:XbaI).LEU2* | This Study |
