# Supplementary material for: Functional Centromeres Determine the Activation Time of Pericentric Origins of DNA Replication in Saccharomyces cerevisiae
Source: PLoS Genet. 2012 May 10;8(5):e1002677. doi: 10.1371/journal.pgen.1002677 (PMC3349730; doi:10.1371/journal.pgen.1002677)
Supplement: Table S2 — Primers used during strain construction. (DOC) [file pgen.1002677.s012.doc]

Supplemental Table S2. Primers

| Primer Number | Sequence | Use |
| --- | --- | --- |
| 71 | CCAATAGATGAGCTTCCGCTT | Hybridizes upstream of *MET2*. Used to confirm integration of *CEN7* and *LEU2* at the *MET2* locus. Also, to clone the 5’ half of *met2::CEN7.LEU2* to pUC18-*KanMX-ARS228* vector. |
| 72 | GTGCGGTCAAAATGTGGAAA | Hybridizes downstream of *MET2*. Used to confirm integration of *CEN7* and *LEU2* at the *MET2* locus. Also to clone the 3’ half of *met2::CEN7.LEU2* to pUC18-*KanMX-ARS228* vector. |
| 88 | TGGCAAAACGACGATCTTCT | Hybridizes in the 3’ end to *LEU2*. Used to confirm integration of *CEN7* and *LEU2* at the *MET2* locus. |
| 126 | ATCGCCACTATCTTGTCTGCT | Hybridizes in the 5’ end to *LEU2*. Used to confirm integration of *CEN7* and *LEU2* at the *MET2* locus. |
| 133 | TTGGCCTCTTCAAGATTATGG | Hybridizes downstream of the EcoRV site in *LEU2*. Used to clone the 5’ half of *met2::CEN7.LEU2* to pUC18-*KanMX-ARS228* vector. |
| 134 | TGGCGATAGGGTCAACCTTAT | Hybridizes upstream of the EcoRV site in *LEU2*. Used to clone the 3’ half of *met2::CEN7.LEU2* to pUC18-*KanMX-ARS228* vector. |
| 147 | ATTTTTGTTTTTGCCTTCTAGAAAGAAAATAGTTTTTTGTATTACGG | Hybridizes to *CEN7*. Used to mutate CDEIII in pTP18 via site directed mutagenesis. |
| 148 | CCGTAATACAAAAAACTATTTTCTTTCTAGAAGGCAAAAACAAAAAT | Hybridizes to *CEN7*. Used to mutate CDEIII in pTP18 via site directed mutagenesis. |
